# Olive Oil as a Potential Source of Anticancer Phytochemicals: In Vitro and Preclinical Evaluation of Active Ingredients and Their Synergism with Conventional Treatments

**DOI:** 10.3390/nu18213466

**Published:** 2026-10-22

**Authors:** Wamidh H. Talib, Intisar Hadi Al-Yasari, Jomana Alqannas, Shahd Mismar, Doaa Anbarserry, Wafa` A. Ahmad, Douglas Law, Ahmad Riyad Alsayed, Bence Raposa

**Affiliations:** 1 Faculty of Allied Medical Sciences, Applied Science Private University, Amman 11931, Jordan; 2 Department of Genetic Engineering, College of Biotechnology, Al-Qasim Green University, Babylon 51013, Iraq; 3 Department of Clinical Nutrition and Dietetics, Applied Science Private University, Amman 11931, Jordan; 4 Department of Clinical Pharmacology, Faculty of Medicine, King Abdulaziz University, Rabigh 21589, Saudi Arabia; 5 Department of Basic Science, Faculty of Science, Applied Science Private University, Amman 11931, Jordan; 6 Faculty of Health and Life Sciences, Inti International University, Nilai 71800, Malaysia; 7 Department of Clinical Pharmacy and Therapeutics, Applied Science Private University, Amman 11931, Jordan; 8 Institute of Basics of Health Sciences, Midwifery and Health Visiting, Faculty of Health Sciences, University of Pécs, 7621 Pécs, Hungary

**Keywords:** cancer, Mediterranean diet, olive oil, phenolic compounds

## Abstract

Cancer is a widespread disease and a leading cause of death worldwide. This has increased interest in foods that contribute to lowering its incidence. Olive oil is a key component of the Mediterranean diet and contains numerous bioactive compounds, including oleuropein, hydroxytyrosol, oleocanthal, squalene, sterols, tyrosol, and oleacein, which possess various properties and benefits. This review examines the potential of these compounds as anticancer agents and their ability to manage various types of cancer. Chemical structure, mechanisms of action, and potential to work in combination with conventional treatments were discussed for each compound. Studies discussed in this review show promising anticancer effects of olive oil compounds with high potential to augment conventional treatments through the anticipated anticancer effect of the Mediterranean diet with olive oil. This review focused on in vitro and preclinical studies of using olive oil phytochemicals in cancer research. However, further clinical and experimental studies are needed to determine the optimal dosage of olive oil and better confirm its therapeutic efficacy.

## 1. Introduction

Cancer remains a major health problem worldwide, ranking 2nd as the leading cause of death worldwide, after cardiovascular diseases. There are over 130 types of cancer that have been found so far, with breast, lung, colorectal, prostate, and stomach cancers being among the most common. The incidence of new cases of cancer is expected to increase dramatically over the next 16 years, from 19.3 million cases in 2024 to about 28.4 million cases in 2040 [1]. It is estimated that in 2018, 9.6 million people died from cancer [2].

Carcinogenesis is driven by molecular changes, leading to the accumulation of genetic changes that dysregulate cell death and growth. Such changes may be inherited or induced by exposure to oncogenic viruses, ionising radiation or chemical agents [3]. Other risk factors for cancer include unhealthy eating, obesity, excessive alcohol intake, smoking, and low levels of some of the micronutrients that help prevent cancer [4]. Traditional cancer therapies include surgery, chemotherapy, radiation, and targeted molecular therapies and have improved survival rates but are often associated with significant adverse effects, such as impaired immune function and secondary cancers [3,5,6].

Under these restrictions, the health benefits of dietary intervention have been scientifically proven to prevent cancer. A healthy diet and physical activity can help to prevent up to 40% of cancers [4]. The dietary pattern is directly related to human epidemiological evidence; people who consume more olive oil or follow the Mediterranean diet tend to show favorable associations. Meta-analyses and cohort/case–control studies indicate that greater adherence to the Mediterranean diet is associated with a lower incidence of cardiovascular disease and related mortality [7]. Olive oil intake is also associated with lower risk of cancer, type 2 diabetes, and some cardiovascular biomarkers [8,9]. A 16% lower risk of type 2 diabetes was observed in the highest olive oil intake category, consistent with indications of a lower risk of developing the disease [10]. Many previous epidemiologic studies found favorable associations with cognitive performance [11]. Whereas the experimental studies that isolated olive compounds can explain those effects and mostly provide plausible mechanisms rather than definitive clinical proof [12]. Compounds mainly provide biological credibility for why olive-rich diets may be beneficial. Hydroxytyrosol and oleuropein repeatedly show antioxidant and anti-inflammatory actions [13], including effects on LDL oxidation [14], mast cell degranulation [15], platelet activation [16], and enzyme inhibition [17]. These compounds can act on molecular pathways relevant to cardiovascular, metabolic, cancer, inflammatory, and neurodegenerative diseases [12,18]. According to the World Cancer Research Fund, olive oil is a traditional food consumed in the Mediterranean and has been suggested to reduce the risk of many types of cancer; several studies support this in Mediterranean populations. Studies indicate that people who follow a Mediterranean diet have lower rates of stomach, esophageal, and lung cancers [19], and an inverse relationship between olive oil use and risk of postmenopausal breast cancer has been identified [20]. A pool of 45 individual studies was used for systematic review and meta-analysis, and overall, the highest olive oil consumption was associated with a 31% reduced risk of cancer compared with the lowest consumption [21]. However, several studies have highlighted that human evidence remains limited, bioavailability is variable, and much of the reported anticancer activity is derived from in vitro and animal models rather than clinical trials [22,23,24,25].

Extra virgin olive oil (EVOO), an essential part of the Mediterranean diet, is a rich source of bioactive polyphenols, including hydroxytyrosol, oleuropein, oleocanthal, oleacein, tyrosol, and squalene [20]. These compounds have been shown to affect critical biological processes that are important to cancer chemoprevention and treatment strategies, including immunomodulation, anti-inflammatory effects, cell proliferation, apoptosis, angiogenesis, and metastasis [20]. All polyphenols have the potential to influence every stage of chemoprevention: primary prevention (preventing cancer initiation); secondary prevention (preventing cancer promotion); and therapeutic intervention (preventing cancer progression) [26]. Studies have shown that olive polyphenols—such as hydroxytyrosol and oleuropein—exert beneficial effects, including reducing the harmful side effects of treatment, enhancing chemotherapy effectiveness, and inhibiting the proliferation of tumour cells [20]. Several Preclinical studies suggest isolated phytochemicals from olive oil enhance chemotherapy efficacy and may modulate cancer-related pathways through in vivo and mechanistic studies but do not support established clinical chemotherapy benefit [27,28].

The doses and concentrations investigated for the anticancer effects of EVOO and its bioactive constituents vary depending on the compound, formulation, and experimental design [29,30,31,32]. In vitro studies have commonly evaluated isolated phenolic compounds at micromolar concentrations, with values of approximately 1–100 μM reported in several models [31,33], although concentrations outside this range have also been studied [34]. In animal studies, selected compounds have been administered at doses expressed in mg/kg body weight [35,36,37]; even human dietary interventions have generally involved daily EVOO consumption, often in the range of 20–50 g or mL [38,39]. Direct comparisons between these approaches are limited by differences in phenolic composition, formulation, and bioavailability, particularly when distinguishing whole EVOO from purified compounds and phenolic-enriched extracts [24,40]. Furthermore, human studies examining cancer-related effects have primarily focused on intermediate biomarkers [41], including oxidative DNA damage and other biomarkers potentially associated with cancer risk, rather than clinical outcomes such as cancer incidence, disease progression, or survival [42,43,44]. Therefore, evaluating the doses and concentrations used across in vitro, animal, and human studies is essential for interpreting the available evidence, recognizing its limitations, and identifying the research needed to establish the clinical relevance of EVOO-derived compounds in cancer prevention and treatment [25,29,32,38,45,46].

This review studies the bioactive compounds of EVOO and their potential anticancer effects, with particular emphasis on oleuropein, hydroxytyrosol, oleacein, oleocanthal, squalene, and tyrosol. The review focuses on their chemical characteristics, biological activities, and the molecular mechanisms through which they may affect cancer-related processes, including cell proliferation, apoptosis, angiogenesis, migration, invasion, and metastasis. The available evidence from in vitro, in vivo, and human studies was considered separately to provide a clearer understanding of the level of evidence supporting the reported effects. A literature search was conducted using PubMed/MEDLINE, Scopus, Web of Science, and Google Scholar for publications since 2000. The search strategy combined terms related to olive oil and its bioactive constituents with cancer-related terms. These included “olive oil,” “extra-virgin olive oil,” “EVOO,” “phenolic compounds,” “polyphenols,” and the individual compound names, together with terms such as “cancer,” “anticancer activity,” “apoptosis,” “oxidative stress,” “angiogenesis,” “metastasis,” and “molecular mechanisms.” Combinations of these terms were used to identify relevant studies across the databases. A study was included in the review if it had been peer-reviewed and if it had supplied original or relevant evidence regarding the biological or anticancer effects of the compounds of interest. Special emphasis was placed on primary research articles that reported original results from in vitro, in vivo, or human/clinical studies. Where available, studies that used purified individual compounds were differentiated from those that used EVOO, extracts from olive fruit or leaves, or other complex preparations. This distinction was seen as important since the biological effects seen with a purified compound cannot always be attributed to EVOO or to the whole of an olive-derived extract. Primary studies were prioritized for reporting specific experimental findings, such as anticancer effects, concentrations, doses, molecular responses, and treatment outcomes. The evidence was subsequently organized according to the experimental setting: in vitro/cell-based studies, in vivo/animal studies, and human/clinical studies. This approach was used to avoid conflating findings obtained from different levels of evidence. Where clinical evidence was unavailable or limited for a particular compound, this limitation was explicitly indicated rather than extrapolating findings from cellular or animal models to human efficacy. The extracted information included the phytochemical or preparation investigated, experimental model, cancer type, treatment concentration or dose, duration of exposure, principal biological or anticancer outcome, reported molecular mechanisms, and study type. This information was used to provide a structured comparison of the available evidence and to facilitate a critical assessment of the concentrations and doses reported across experimental models. Review articles and systematic reviews were used selectively to provide related information, summarize established data, and identify relevant primary studies but were not considered evidence for specific experimental findings.

## 2. Chemical Composition of Olive Oil

Olive oil is a vegetable oil widely consumed in the Mediterranean diet due to its health benefits, particularly its polyphenol content. Evidence suggests a lower risk of various cancer types among Mediterranean populations, where olive oil is a staple traditional food and a primary source of plant-based fats [21]. Olive oil consists of up to 99% triglycerides, with oleic acid being the main component, accounting for up to 83% of total fatty acids. It is typically categorized into two main fractions: the saponifiable and the unsaponifiable fraction. The saponifiable fraction constitutes over 98% of the total composition, primarily of triglycerides rich in monounsaturated fatty acids—specifically oleic acid (55–83%)—alongside smaller amounts of saturated fatty acids, such as palmitic acid (7.5–20%), and polyunsaturated fatty acids, such as linoleic acid (3.5–21%) [47]. As the predominant monounsaturated fatty acid, oleic acid is largely responsible for the health benefits associated with the fat content type of olive oil, including its cardioprotective and anti-inflammatory properties [48].

**Figure 1:**
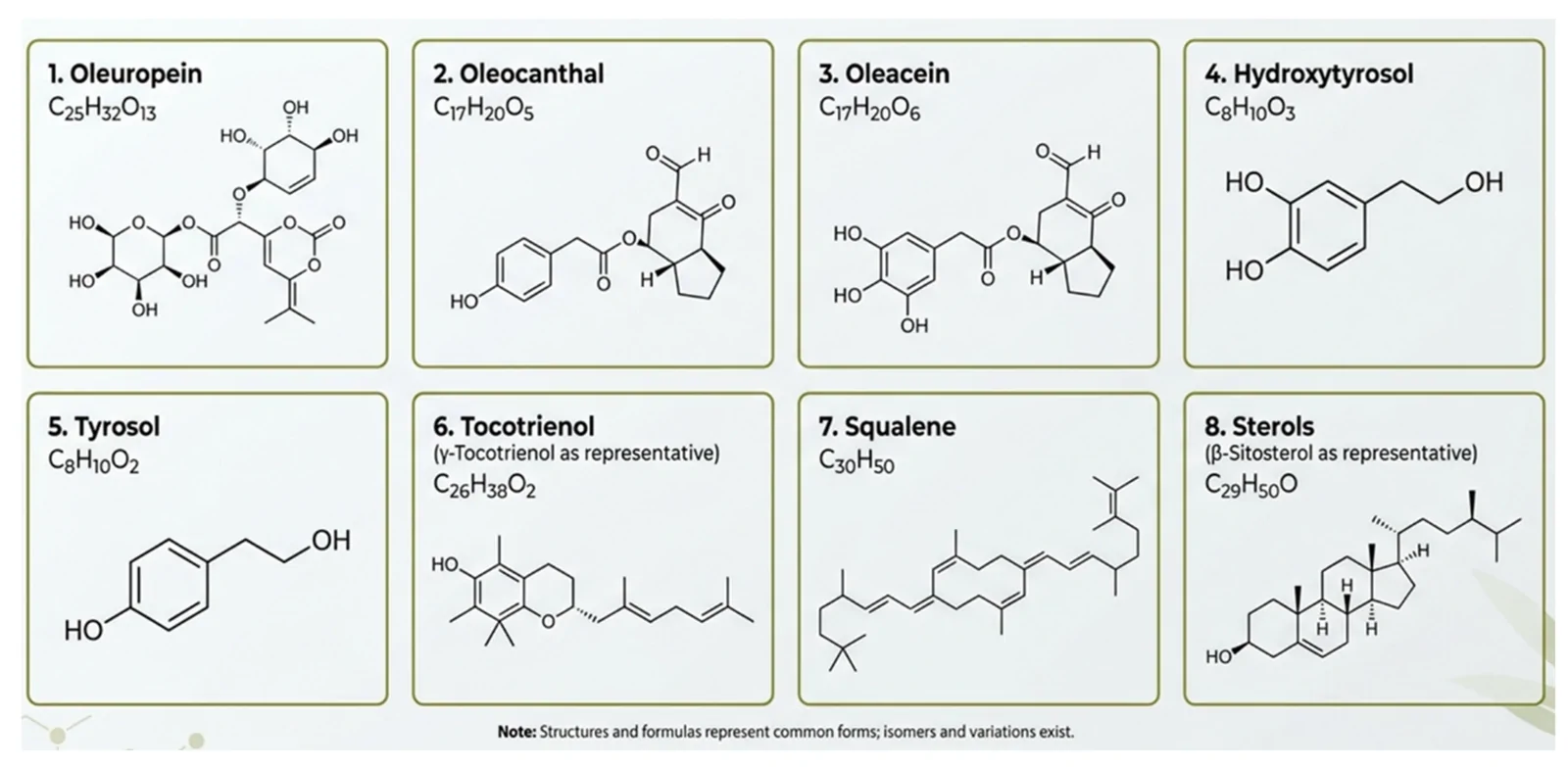
Chemical structures of major bioactive compounds present in EVOO, including oleuropein, oleocanthal, oleacein, hydroxytyrosol, tyrosol, tocotrienols, squalene, and sterols. The chemical structures were retrieved from PubChem compound records and redrawn by the authors.

In contrast, the unsaponifiable fraction, although representing less than 2% of the total composition, contains a diverse and biologically important array of bioactive compounds, including sterols, tocopherols, phenolic alcohols, secoiridoids, pigments, and hydrocarbons such as squalene. Despite their low concentration, these secondary compounds are considered the main contributors to the distinctive biological properties and health benefits of EVOO. Olive oil derives its benefits from its constituents—including phenolic compounds and secondary metabolites—which contribute to body protection and regulate inflammatory responses. These compounds are characterized by potent antioxidant, anti-inflammatory, and anti-cancer properties, as well as the ability to modulate immune responses, thereby protecting molecules from oxidative damage. Key compounds include hydroxytyrosol, tyrosol, oleocanthal, oleacein, and oleuropein; these substances significantly inhibit cancer cell growth, highlighting their potential role in cancer prevention [20,21,47,49,50,51]. The major chemical constituents of olive oil, including both the saponifiable and unsaponifiable fractions, along with their principal components and typical concentration ranges, are summarized in Table 1. The chemical structures of the major bioactive compounds discussed in this review are presented in Figure 1.

## 3. Bioactive Compounds and Anticancer Mechanisms of Olive Oil

### 3.1. Oleuropein

#### 3.1.1. Chemical Composition of Oleuropein

Oleuropein, the compound that is most abundant in all parts of the olive tree *Olea europaea* L., is present at 14–19% in olive leaves and 2.28 mg/g of hydroxytyrosol (3,4-dihydroxyphenylethanol, HT) as its main degradation derivative, especially in raw olive fruits and leaves. Oleuropein is a secoiridoid glucoside consisting of hydroxytyrosol esterified to elenolic acid, which was first identified in 1908. It is found in EVOO at 12–150 mg/kg and up to 90 mg/g in olive leaves [19,20]. It belongs to the secoiridoid class and is part of the unsaponifiable fraction of EVOO, a minor fraction but rich in bioactive compounds such as sterols, tocopherols, phenolic alcohols, secoiridoids, pigments, and hydrocarbons [47]. Oleuropein and hydroxytyrosol derivatives are also present in the fruit and oil of *Olea europaea* L. and therefore can be easily ingested as part of a regular diet. However, they may also be available from other sources, such as olive mill wastewater [52].

#### 3.1.2. Health Benefits of Oleuropein and Anti-Cancer Properties

Oleuropein has been studied for its potential health benefits [52]. Research in this area has examined its effects using different experimental approaches, ranging from in vitro and in vivo experimental models to human studies. Particularly, its postprandial glucose regulation and insulin sensitivity. In a randomized crossover study involving 20 healthy subjects, a single 20 mg dose of oleuropein before a meal reduced postprandial blood glucose and oxidative stress markers and altered DPP-4 and GLP-1 responses, but the acute study does not establish long-term diabetes prevention or treatment [53]. Additionally, a 2025 randomized trial in 40 older men found an approximately 25% increase in fractional pyruvate dehydrogenase activity after 36 days of supplementation providing 100 mg oleuropein daily. LDL cholesterol was also lower, but muscle strength, fatigue, mitochondrial respiration, and whole-body metabolism did not improve significantly [54].

In vitro studies have investigated the effects of oleuropein on cancer cell proliferation, apoptosis, and molecular signaling pathways involved in tumour development and progression. Concentration, exposure time, and cancer type explain different processes of oleuropein and its effects on cancer cells [19]. The anti-proliferative effect of oleuropein has been demonstrated in MCF-7 cell lines by a statistically significant block at the G1/S phase transition and an increase in the number of cells in the G0/G1 phase [55]. Oleuropein reduced cell viability in MDA-MB-231 cells, increased ROS production, inhibited cell migration and invasion, and suppressed NF-κB activation [56]. Oleuropein plays a key role as a pro-oxidant and an antioxidant in cancer. For the first time, a previous study confirmed that high doses of oleuropein showed anti-proliferative and pro-apoptotic activity in MCF-7 breast cancer and HEY ovarian cancer cells. Moreover, the results indicate that low doses of oleuropein impair cell viability without affecting cell mortality. Interestingly, oleuropein decreases intracellular levels of ROS and LIP in ovarian cancer cells treated with erastin and increases the GPX4 scavenging enzyme, with a consistent reduction in mitochondrial ROS [29]. Moreover, Oleuropein dose-dependently reduced the viability of TCAM-2 and SEM-1 seminoma cells, decreased cyclin D1 expression, and increased p21 and BAX expression while reducing nuclear NF-κB levels. It also induced apoptosis and inhibited cell migration and motility, accompanied by downregulation of TGF-β1. Oleuropein dose-dependently inhibited HepG2 hepatocellular carcinoma cell viability and induced apoptosis through caspase activation, modulation of BAX and Bcl-2 expression, and suppression of AKT signaling. It also increased reactive oxygen species (ROS) production, suggesting that its anticancer effects are partially ROS-dependent [57]. Oleuropein reduced the viability of U251 and A172 glioma cells and induced apoptosis. Bax is proapoptotic, and Bcl-2 is antiapoptotic. Bax and Bcl-2 have been shown to induce apoptosis in glioma cells by upregulating caspase-3 and 9. It also inhibited cell migration and invasion by suppressing AKT phosphorylation and downregulating MMP-2 and MMP-9. Furthermore, the PI3K inhibitor LY294002 enhanced its pro-apoptotic and anti-invasive effects, supporting the involvement of the PI3K/AKT signaling pathway in its anticancer activity [58]. Oleuropein at 370 µM showed anti-metastatic effects in the MDA-MB-231 cell line; this effect is due to the increased tissue suppression of the activity of the metalloproteinases, increasing the apoptosis rate and inhibiting the activity of the matrix metalloproteinases which stimulate metastasis from tumour cells [19].

An olive oil polyphenolic extract containing hydroxytyrosol, tyrosol, and several secoiridoids, including oleuropein, reduced COX-2 expression, inhibited p38 and CREB phosphorylation, and induced G2/M cell-cycle arrest in human colon adenocarcinoma cells [59]. However, because a mixed extract was used, these effects cannot be attributed precisely to oleuropein. In another study using isolated oleuropein, treatment of HT-29 colorectal cancer cells inhibited cell proliferation and modulated several cancer-associated molecular responses, including downregulating HIF-1α expression. Notably, hydroxytyrosol, rather than oleuropein, significantly increased PPARγ expression in this model [60]. Furthermore, in a mouse model of colitis-associated colorectal cancer, oleuropein reduced colonic tumor growth and downregulated COX-2, NF-κB, Wnt/β-catenin, PI3K/Akt, and STAT3 signaling pathways [61].

Furthermore, it disrupted proliferation, migration, and cytoskeletal integrity [62], as well as tumor growth, often alongside altered oxidative stress markers [35]. Pro-oxidant-like effects have been shown in some tumor settings or Fenton-like conditions, where oleuropein can participate in redox cycling or selectively push cancer cells toward oxidative stress and death in K562 leukemia cancer cells [63]. Oleuropein exhibited antioxidant activity that is attributed, in part, to chelating metal ions (copper and iron), which act as catalysts for the formation of free radicals, and inhibition of several enzymes associated with inflammation. The antioxidant activity of oleuropein is dose-, time-, and cell type-dependent. At concentrations above 100 µmol/L, cells were killed; at lower concentrations, cells were protected against damage by antioxidants. Antioxidant effects were shown in the non-malignant tissues, while pro-oxidant stimulation of ROS activation was observed in cancer tissues like breast and prostate cells and leukemia HL-60 cells [19]. One of the main phenolic compounds in olives, oleuropein, has been widely explored for its cancer-fighting activity [20].

In vivo studies have further explored the potential chemopreventive effects of oleuropein in animal models of cancer. Oleuropein has demonstrated anti-cancer activity through both anti-angiogenic and pro-apoptotic actions on a variety of molecular pathways that are known strategies required for tumour growth and survival. Researchers investigated the chemopreventive effects of oleuropein in a mouse model of colitis-associated colorectal cancer. Oleuropein reduced colonic tumour growth and multiplicity, reduced intestinal inflammation, and downregulated cancer-associated signalling pathways, including NF-κB, Wnt/β-catenin, PI3K/Akt, and STAT3 [61]. Interestingly, Oleuropein at doses of 10–100 mg/kg has demonstrated protective effects in animal models, with 50 mg/kg showing marked improvement in oxidative stress, renal function, and apoptotic markers; however, these results do not establish its safety or therapeutic efficacy in humans [64]. Oleuropein could be effective in inhibiting skin carcinogenesis in vivo. Oleuropein at both doses (10 mg/kg and 25 mg/kg) significantly reduced the total volume of tumour-bearing mice, decreased the expression levels of invasion enzymes MMP2, pro-MMP9, and MMP9, decreased the level of tissue colitis-associated colorectal cancer, and decreased the percentage of Ki-67+ cells and PECAM-1 (CD31) + areas on skin in UVB-irradiated albino hairless HOS:HR-1 mice. Oleuropein also seems to be beneficial for preventing melanoma growth and metastasis [30].

**Figure 2:**
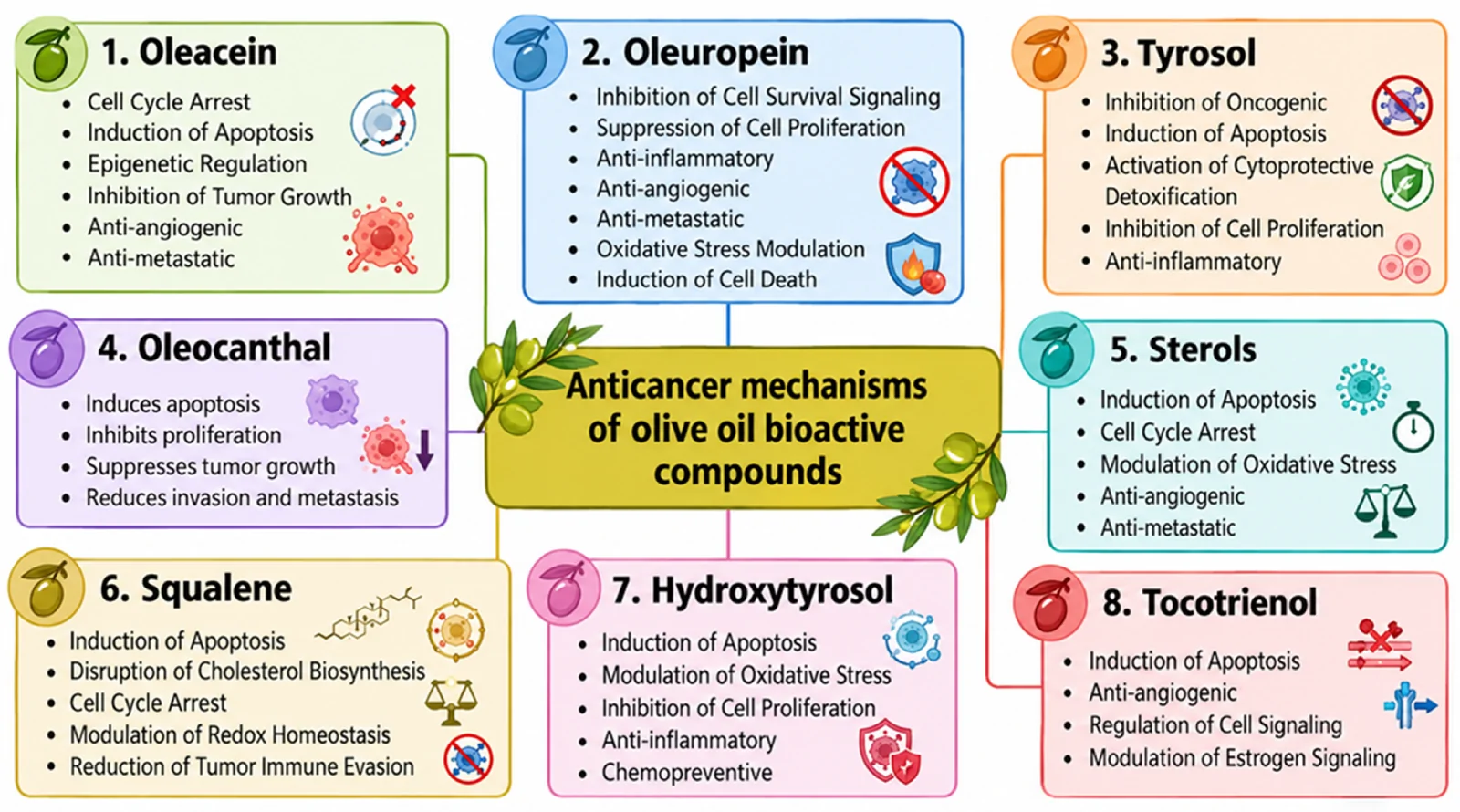
Schematic overview of the principal anticancer mechanisms attributed to olive oil bioactive compounds. These mechanisms include the induction of apoptosis, cell-cycle arrest, modulation of cancer-associated signaling pathways, suppression of inflammation and oxidative stress, and inhibition of angiogenesis, tumor growth, invasion, and metastasis. These effects have been investigated in in vitro and in vivo experimental models, as well as in human studies examining dietary exposure and cancer-related biomarkers. Done by the authors.

In a B16F10 melanoma allograft mouse model, dietary oleuropein inhibited tumor angiogenesis and lymphangiogenesis and suppressed high-fat diet-induced solid tumor growth and lymph node metastasis. Inhibition of angiogenesis and lymphangiogenesis was associated with reduced expression of VEGF-A, VEGF-C, VEGF-D, and their related receptors, as well as reduced accumulation of M2 macrophages and lipid vacuoles in tumor tissues [65]. Oleuropein also prevented increases in MMP-2, MMP-9, MMP-13, VEGF, and COX-2, and reduced Ki-67 and CD31-positive cells, consistent with anti-proliferative and anti-angiogenic effects [65]. Similarly, in a UVB-induced skin carcinogenesis model, oral administration of oleuropein reduced skin tumor development and growth, accompanied by decreased expression of COX-2, VEGF, and matrix metalloproteinases [66]. When used, oleuropein is reported to stimulate autophagy and mitophagy by increasing the number of autophagosomes, promoting clearance of damaged mitochondria, and reducing oxidative stress [66]. Moreover, chronic treatment with oleuropein has been reported to elevate levels of antioxidant enzymes such as superoxide dismutase and glutathione, leading to lower oxidative stress and hypertension in rats [67]. However, these effects were demonstrated in an animal model and require validation in human clinical studies. The anticancer mechanisms of oleuropein and the supporting evidence are illustrated in Figure 2 and summarized in Table 2.

Oleuropein has some human evidence for cardiovascular [68] and inflammatory benefits [69], but no direct human clinical evidence demonstrating anti-cancer efficacy. Also, observational studies suggest that higher olive oil intake is associated with lower overall cancer prevalence or risk, but the available evidence here does not specify cancer sites. A 2022 meta-analysis found lower likelihoods for breast cancer and gastrointestinal cancer with higher olive oil intake, and an inverse association for upper aerodigestive tract and urinary tract cancers [21]. In addition, Phenolic compounds found in EVOO, including oleuropein and hydroxytyrosol, may exhibit prebiotic-like properties by modulating gut microbiota composition and microbial metabolism [70], thereby favouring the growth of beneficial bacteria (probiotics) such as *Bifidobacterium* and inhibiting the growth of pathogenic bacteria such as *Escherichia coli* [47].

### 3.2. Oleocanthal

#### 3.2.1. Chemical Composition of Oleocanthal

The term oleocanthal is derived from “oleo” (olive), “canth” (pungent sensation), and “al” (aldehyde). It was described in the 1990s as a phenolic compound, specifically a carboxyl ester. Oleocanthal is not found, or is present only at low concentrations, in European olive trees. However, it is derived through various enzymatic reactions during the production and processing of EVOO. Oleocanthal has been identified as the component responsible for the pungent taste in olive oil. This pungent sensation when consuming olive oil, especially virgin olive oil, indicates a high concentration of oleocanthal, not a high concentration of polyphenols [71].

#### 3.2.2. Health Benefits of Oleocanthal and Anti-Cancer Properties

Oleocanthal is a bioactive olive oil phenolic with strong preclinical anti-inflammatory and anticancer effects. Oleocanthal reduced ROS and inflammatory mediators in macrophage-like cells and murine macrophages [72] and inhibited NF-κB/MAPK/inflammasome-related signaling in vitro [73]. A human crossover trial in obesity and prediabetes also found improved inflammatory and antioxidant status with oleocanthal-rich EVOO [74]. Oleocanthal has significant potential to treat cancer and inflammation by inhibiting cancer cell viability and spread, inducing apoptosis, and mitigating the side effects of radiotherapy and chemotherapy, thereby enhancing their effectiveness [75]. Oleocanthal shows preclinical anticancer activity against hepatocellular carcinoma (HCC) through inhibiting cancer cell proliferation and cell-cycle progression while inducing apoptosis. It also reduced cell migration and invasion, suppressed tumor growth in an orthotopic HCC model, and impeded lung metastasis in vivo. Mechanistically, these effects were associated with inhibition of STAT3 signaling and epithelial–mesenchymal transition (EMT) through the downregulation of Twist. Oleocanthal also reduced the expression of STAT3 downstream effectors, including Cyclin D1, Bcl-2, survivin, and MMP-2, partly through modulation of JAK1/JAK2, SHP-1, and Hsp90 pathways [76]. Oleocanthal may exert anticancer effects by disrupting lysosomal membrane integrity, thereby promoting cancer cell toxicity [77].

Oleocanthal is found in high concentrations in olive oil, especially EVOO, compared to regular oil. This oil contributes to alleviating inflammatory diseases, arteriosclerosis, cancer, and neurological diseases. The concentration of oleocanthal in olive oil varies, ranging from 0.2 to 498 mg/kg. This is due to the olive varieties, the conditions in which the olive oil grows and matures, as well as the agricultural techniques used and the processing methods. It is also affected by the amount of irrigation and the three-stage centrifugation process, which reduces the amount of oleocanthal. Therefore, researchers suggest using it as a dietary supplement to promote its consumption in a concentrated and regulated dose, which may contribute to the prevention and treatment of some diseases [75].

The body contains a pathway called HGF-c-Met, which is important for regulating cell growth and movement. However, in cancer, this pathway becomes overactive and contributes to disease progression. When HGF binds to the C-Met receptor, abnormal cell growth increases, enhancing cell mobility and proliferation. Cells become more resistant to apoptosis, and new blood vessels form to nourish the tumor, leading to cancer metastasis. Oleocanthal inhibits HGF/c-Met signaling and downstream pathways such as Akt and MAPK/ERK [46], inhibiting several proteins responsible for cancer cell growth, such as protein kinase B (PKB), mitogen–activated protein kinase (MAPK), signal transducers and transcription activators -3 (STAT-3), and mechanistic target of rapamycin (mTOR) [75], thereby reducing proliferation, invasion, migration, angiogenesis, and tumor growth [46].

It also plays an effective role in targeting skin, breast, prostate, pancreatic, and hepatocellular carcinoma, colorectal cancer, lung cancer, and malignant blood tumors such as multiple myeloma and chronic lymphocytic leukemia [71]. Researchers found that treatment with oleocanthal—at low concentrations in the micromolar range—was capable of inhibiting breast cancer growth, limiting cell migration and invasion (in a dose-dependent manner), and halting cell cycle progression from the G1 phase to the S phase [71]. The main anticancer mechanisms associated with oleocanthal are illustrated in Figure 2 and summarized in Table 2.

In animal models, oleocanthal reduced tumor growth and lung metastasis. These effects were associated with the inhibition of STAT3 signaling and the downregulation of target genes involved in cell survival, invasion, and angiogenesis, including Mcl-1, Bcl-xL, MMP-2, MMP-9, and VEGF [78]. In a genetically engineered mouse model of pancreatic neuroendocrine tumors, oleocanthal treatment reduced tumor burden and prolonged survival [79]. These findings suggest that oleocanthal may have potential as an anticancer compound; however, further studies are needed to establish its efficacy and safety in humans.

Human evidence is mainly about health-related effects rather than direct anticancer treatment: an oleocanthal/oleacein-rich EVOO improved inflammatory and oxidative status in people with obesity and prediabetes, and an oleocanthal-rich oil showed acute antiplatelet effects in type II diabetes [74,80]. A pilot randomized trial in early CLL reported favorable changes in blood and apoptosis/proliferation markers with high-oleocanthal/oleacein EVOO [38].

### 3.3. Oleacein

#### 3.3.1. Chemical Composition of Oleacein

Oleacein is a critical bioactive secoiridoid polyphenol and one of the most abundant secoiridoids found in EVOO. Although it has specific biological activities, it shares structural similarities with other studied olive polyphenols, like oleuropein and oleocanthal [20]. It is a phenylethanoid ester of hydroxytyrosol and a secoiridoidic acid and thus is part of the unsaponifiable fraction of EVOO, as well as other secoiridoids such as oleuropein and ligstroside derivatives [47,81]. The polyphenolic fraction of extra virgin olive oil consists of simple phenols (tyrosol and hydroxytyrosol), secoiridoids (oleuropein, oleocanthal, and oleacein) and lignans, all of which contain elenoic acid or derivatives as part of their molecular structure [81].

#### 3.3.2. Health Benefits of Oleacein and Anti-Cancer Properties

Oleacein has recently been found to possess several beneficial properties in diverse disease settings, for which its strong anti-inflammatory, anticancer, and antioxidant activities are gaining interest [20]. The bioactive compounds that are present in EVOO, in particular phenolic compounds, such as oleuropein, hydroxytyrosol, oleocanthal, and oleacein, have a system-wide effect on inflammation—from the signaling pathways that trigger inflammation to cytokines that induce inflammation to the immune system as a whole [47]. Anticancer activity of single phenols, particularly hydroxytyrosol, tyrosol, and derivatives oleuropein, oleacein, and oleocanthal, was widely investigated on different types of cancer cells such as colorectal, prostate, hepatocellular, pancreatic, and lung cancer, and the antioxidant activity of phenolic compounds present in olive oil was attributed to the anticancer activity [82]. The anticancer mechanisms of these bioactive compounds and the supporting evidence are illustrated in Figure 2 and summarized in Table 2.

Oleacein inhibits STAT3, activates the apoptotic machinery, and exerts anti-metastatic effects in the SH-SY5Y human neuroblastoma cells. Oleacein can reduce cell proliferation by blocking the cell cycle at the S phase, increasing Bax and p53, and reducing Bcl-2 expression and STAT3 phosphorylation. Moreover, oleacein reduced SH-SY5Y cell adhesion and migration. Overall, these findings indicate that oleacein exerts anti-cancer effects against neuroblastoma cells, suggesting a promising role as a candidate against this type of cancer [83].

Oleacein decreased the viability of several multiple myeloma primary samples and cell lines, even when bone marrow stromal cells were present, but did not affect the viability of healthy peripheral blood mononuclear cells. In addition, oleacein was found to down-regulate a number of class I/II histone deacetylases (HDACs) both at mRNA and protein levels, to induce dose-dependent acetylated histones and α-tubulin accumulation, and to block cell clonogenicity, triggering cell cycle blockade and inducing apoptosis [84].

The multiple activities of oleacein enhance the health benefits of the Mediterranean diet and make it a promising tool in the therapeutic and chemopreventive arsenal, particularly for cancer treatment [20]. The use of synthetic or natural medicines to prevent or delay the development of particular diseases such as cancer occurs via mechanisms of programmed cell death (autophagy and apoptosis), cell growth regulation, and antioxidant and anti-inflammatory properties. Furthermore, these drugs can modulate key signaling pathways, suppress metastasis, inhibit tumour angiogenesis, and inhibit necrosis pathways [20].

However, oleacein has been shown to have potent anti-cancer properties in numerous in vitro studies, including the ability to inhibit critical signaling cascades, disrupt necrosis pathways, prevent tumour angiogenesis, and inhibit metastatic behaviour. Oleacein exhibited antiproliferative activity against 501Mel human melanoma cells, with an IC_50_ in the low micromolar range. Oleacein induced G1/S cell-cycle arrest and DNA fragmentation, downregulated the antiapoptotic genes Bcl-2 and MCL1 and the proliferation-related genes c-KIT, K-RAS, PIK3R3, and mTOR, and increased BAX expression. Oleacein also modulated several microRNAs involved in the regulation of apoptosis and cell proliferation. The results demonstrate the ability of oleacein to contrast the proliferation of cutaneous melanoma cells through the transcriptional modulation of relevant genes and microRNAs [85], confirming the anticancer potential of EVOO and suggesting oleacein as a chemopreventive agent for cancer disease therapy.

Recent in vivo and clinical research suggests that high-phenolic EVOO may be useful in managing prediabetes and obesity by reducing the markers of oxidative stress and inflammation. Oleacein inhibited endothelial cell proliferation, invasion, and tube formation in vitro; it suppressed cell migration and induced apoptosis. Their anti-angiogenic activities were further supported by findings from two in vivo models. Mechanistically, both compounds modulated signaling pathways involved in cell survival and proliferation. These findings suggest that oleacein and oleocanthal may contribute to the potential anticancer properties of extra virgin olive oil (EVOO) by interfering with tumor angiogenesis [86]. However, further research is needed to establish their efficacy and safety in humans.

A randomized clinical trial was conducted for patients with early-stage chronic lymphocytic leukemia; daily consumption of EVOOi, oleacein, and oleocanthal significantly increased apoptotic markers such as cleaved cytokeratin 18 and Fas/Apo-1, and reduced those of survivin and cyclin D [38]. In addition, the results suggest a new anti-aging effect of oleacein, but this requires further investigation, including controlling cell cycles, promoting apoptosis, suppressing angiogenesis, and regulating epigenetics. Despite these promising results, oleacein has not been as extensively researched as its counterparts, such as oleocanthal and oleuropein; therefore, additional preclinical and clinical studies are required to establish its therapeutic potential [20].

### 3.4. Hydroxytyrosol

#### 3.4.1. Chemical Composition of Hydroxytyrosol

Hydroxytyrosol is a simple phenolic alcohol found primarily in olive oil. Its molecular structure consists of a benzene ring bearing two adjacent hydroxyl groups—a configuration known as a “catechol group.” This structure imparts significant biological properties to the compound, most notably its antioxidant activity; the catechol group serves as the primary reactive site for scavenging free radicals [87].

#### 3.4.2. Health Benefit of Hydroxytyrosol and Anti-Cancer Properties

Hydroxytyrosol belongs to the polyphenol group, compounds abundant in olive oil, especially EVOO, and is considered the strongest antioxidant after gallic acid and the most potent phenolic compound extracted from olive oil, followed by oleuropein, caffeic acid, and tyrosol. It possesses antioxidant, anti-inflammatory, and anticancer properties [88]. It is capable of eliminating free radicals and reactive oxygen species (ROS) and activating the body’s own antioxidant systems. Studies have confirmed its effectiveness against various cancers by inhibiting cell death [50]. Hydroxytyrosol exhibited significant in vitro anti-inflammatory activity in LPS-stimulated RAW264.7 murine macrophages. It inhibited nitric oxide (NO) and prostaglandin E_2_ (PGE_2_) production, with IC_50_ values of 11.4 and 19.5 µM, respectively. HT also reduced the secretion of pro-inflammatory cytokines (IL-1α, IL-1β, IL-6, IL-12, and TNF-α) and chemokines, and downregulated inflammatory genes partly through modulation of the NF-κB signaling pathway. Hydroxytyrosol has been described as one of the most potent anti-inflammatory compounds among olive oil polyphenols [89]. Hydroxytyrosol shows preclinical pro-apoptotic and anti-proliferative activity against multiple cancer cell types; it reduced viability in hepatocellular carcinoma cells [90], thyroid cancer cells, breast cancer cells, colon cancer cells, leukemia cells, glioma cells, melanoma models, and pancreatic cancer [91]. It increased CASP3 and the BAX: Bcl-2 ratio in colorectal cancer cells and reduced proliferation. It also triggered apoptosis in prostate cancer cells [92], with caspase activation, PARP cleavage, and BAX/Bcl-2. Reported mechanisms include intrinsic apoptosis, cytochrome c release, caspase-3 activation, ERK1/2 inhibition, AKT/NF-κB suppression [90], ROS/superoxide generation, and autophagy-related effects [93].

It can eliminate free radicals and reactive oxygen/nitrogen species and activate the body’s endogenous antioxidant systems. Studies have confirmed its effectiveness against various cancer types in inhibiting and killing cells. It also enhances the expression of an antioxidant enzyme. Olive oil phenolic compounds demonstrated protective effects against hydrogen peroxide-induced DNA damage in human peripheral blood mononuclear cells (PBMCs) and HL60 promyelocytic leukemia cells in vitro. Hydroxytyrosol and phenolic extracts from EVOO reduced DNA damage at concentrations as low as 1 µM. At 10 µM, hydroxytyrosol provided 93% protection in HL60 cells and 89% in PBMCs [94]. In addition to its properties as an antidepressant, anti-inflammatory, and anti-atherosclerotic agent, it focuses on regulating oxidative stress within cells by mitigating its intensity, maintaining cellular balance, and reducing the risk of cancer initiation. It plays an important role in modulating inflammatory pathways and arresting the cell cycle, thus stopping uncontrolled cell proliferation [95].

Studies evaluating its role as an anticancer agent against prostate cancer cells without affecting normal cells have shown that it induces apoptosis in prostate cancer cells and inhibits the expression of the androgen receptor, which is present within prostate cells and responds to male hormones, thus helping cancer cells to grow and multiply. Furthermore, it plays a role in modulating inflammatory pathways. Chronic inflammation promotes cancer cell growth and spread, while hydroxytyrosol, through its anti-inflammatory properties, inhibits inflammatory signaling molecules, thereby reducing cancer-promoting processes and mitigating its impact [96].

Hydroxytyrosol plays a significant role as an anticancer agent by reducing the level of the protein PIN1, which is crucial for regulating cell growth. When PIN1 levels decrease, it leads to the translocation of cyclin D1 into the cytoplasm, resulting in its degradation. Cyclin D1 plays a vital role in the cell cycle, facilitating the transition from the G1 to the S phase, thus promoting the growth and proliferation of cancer cells. When pin1 levels decrease, the stability of cyclin D1 within the cell is reduced due to the interaction between pin1 and cyclin D1. This interaction is produced when the levels of peptidyl-prolyl cis-trans isomerase enzymes, NIMA-interaction 1 (pin1), are decreased. This disruption of normal regulation causes cyclin D1 to translocate into the cytoplasm, where it undergoes degradation, preventing its accumulation and consequently inhibiting cancer cell growth [95].

In vitro studies show that hydroxytyrosol enhanced the effects of paclitaxel and cetuximab in breast and colon cancer models [97], and nanoformulation and combination delivery increased anticancer potency in colon and pancreatic cancer cells [98]. The supporting evidence is illustrated in Figure 2 and summarized in Table 2.

### 3.5. Tyrosol

#### 3.5.1. Chemical Composition of Tyrosol

Tyrosol is a simple phenolic alcohol and one of the basic phenolics of olive oil; it is found in the EVOO and table olives [99]. It is present in the unsaponifiable fraction, at a level of mg/kg, together with hydroxytyrosol [47]. Olive oil contains a saponifiable fraction (98–99%), comprising triacylglycerols like oleic, linoleic, palmitic, and stearic acids, and an unsaponifiable fraction (1–2%) composed of hydrocarbons such as squalene, other hydrocarbons, phospholipids, sterols, and vitamins including tocopherols and polyphenols, the latter consisting mainly of tyrosol, hydroxytyrosol, and oleuropein [99]. Hydroxytyrosol is a catechol derivative of tyrosol and is one of the strongest natural radical scavengers and metal chelators [20]. Other important factors contributing to the biological properties of olive oil are minor phenols, such as hydroxytyrosol, tyrosol, and their derivatives of oleuropein, which are believed to have antioxidant and anti-inflammatory properties, among others [47].

#### 3.5.2. Health Benefits of Tyrosol and Anti-Cancer Properties

Tyrosol could reduce damage caused by ROS by induction of antioxidant enzymes [99]. EVOO is rich in phenolic compounds with remarkable biological activities, and some of these, such as tyrosol, hydroxytyrosol, oleuropein, oleacein, and oleocanthal, are of particular interest due to their powerful antioxidant, immunomodulatory, anti-inflammatory, and anticancer effects [20]. Polyphenols are linked to the maintenance of homeostasis in the intestinal epithelium, which contributes to the prevention of tumor development; furthermore, the anti-cancer activity is attributed to the antioxidant properties of the phenolic compounds found in olive oil [82]. Tyrosol could be a protection mechanism for the cells against ROS through activation of antioxidant enzymes and inhibition of oxidative damage [99]. Likely, polyphenols contribute to the maintenance of intestinal epithelium homeostasis, which is important for preventing carcinogenesis, as well as the anti-cancer effect associated with the antioxidant effect of phenolic compounds found in olive oil [82]. Hydroxytyrosol and tyrosol are phenolic compounds that have been associated with antioxidant and anti-inflammatory activities and with the improvement of lipid metabolism, insulin sensitivity, and vascular function [47]. According to an in vitro study. Oleuropein and hydroxytyrosol exhibited anticancer activity against pancreatic cancer cells (MIA PaCa-2), inducing apoptosis through caspase-3/7 activation and changes in Bcl-2 family proteins. Oleuropein showed selective cytotoxicity toward cancer cells while protecting non-tumorigenic pancreatic cells (HPDE), whereas hydroxytyrosol affected both cell types. Gene expression analysis suggested the involvement of c-Jun and c-Fos in apoptosis induction. Tyrosol did not demonstrate comparable activity in the reported findings [100].

Tyrosol inhibits the HIF-1α/NF-κB pathway. It also suppresses the release of pro-inflammatory molecules, such as TNF-α and IL-6—which promote cancer progression—thereby suppressing inflammatory responses within the cancer microenvironment [20,101]. Furthermore, tyrosol inhibits the PI3K/Akt/mTOR/S6K axis and reduces the expression of HIF-1α and its downstream targets. It also has low affinity for the aryl hydrocarbon receptor in the cytoplasm. This causes reduced transcription activity and thus inhibits tumour formation under hypoxic conditions [20]. Apoptosis induced by tyrosol is associated with various key protein changes: an increase in p53 and a decrease in Bcl-2, leading to reduced anti-apoptotic signalling and ultimately cell death. Tyrosol also protects HaCaT keratinocytes from UVB-induced apoptosis, indicating a protective effect in non-neoplastic epithelial cells, and inhibits tumour proliferation [20].

Tyrosol was shown to induce expression of the second-phase detoxification enzyme NAD(P)H: quinone oxidoreductase-1 (NQO1) through the antioxidant response element and suppress the increase in SMMC-7721 human liver cancer cells [20,26]. In addition, tyrosol can help protect cells from ROS by stimulating antioxidant enzymes or preventing oxidative damage, thus confirming its chemopreventive activity in various cancer types [20,102].

Tyrosol demonstrated antitumor activity against colorectal cancer (CRC) in vivo and selectively inhibited CRC cells without disturbing intestinal epithelial cells in vitro. It reduced ROS and inflammatory cytokine levels in MC38 cells and inhibited the activation of the HIF-1α/NF-κB signaling pathways. The study also recognized a relationship between tyrosol and *Faecalibacterium prausnitzii*, a gut bacterium with anti-CRC properties, suggesting that microbial metabolites may contribute to the regulation of tumor growth and inflammation [37].

### 3.6. Squalene

#### 3.6.1. Chemical Composition of Squalene

Squalene belongs to the group of hydrocarbons in the unsaponifiable fraction of EVOO, together with n-alkanes, which are contained in the EVOO in concentrations of mg/kg [47]. It is the most abundant compound in the unsaponifiable matter, making up more than 90% of the hydrocarbon fraction, and it is the last metabolite formed before the sterol ring formation, with a concentration ranging from 200 to 7500 mg/kg oil, in some studies even up to 12,000 mg/kg oil [49]. The amount of squalene can vary with different factors such as the olive growing method and the oil extraction method used [49], and is considerably reduced during the refining stage, thus being found in higher concentrations in extra virgin olive oil and virgin olive oil. Dietary intake (mainly olive oil) and endogenous cholesterol metabolism contribute to the generation of serum squalene [99]. The endogenous synthesis of squalene begins with the generation of 3-hydroxy-3-methylglutaryl coenzyme A (HMG-CoA), the key reduction step of which is niacin-dependent, and the production of mevalonate. Squalene is a major component of human sebum—possessing antioxidant properties—and plays an important role in maintaining skin health [103].

#### 3.6.2. Health Benefits of Squalene and Anti-Cancer Properties

Squalene is a significant antioxidant component of human sebum and has been suggested as a key to maintaining healthy skin; because of its high squalene content, olive oil may be a preventative against skin cancer [99]. The beneficial effects of olive oil may be due to its unique composition. Squalene is characterized by a high content of monounsaturated fatty acids, squalene, and phenolic compounds such as simple phenols, secoiridoids, and lignans, which have high antioxidant activity, helping to reduce oxidative stress and damage to DNA in the cells and to modulate important signaling pathways involved in the development of cancer [21].

Several of the health benefits of olive oil are associated with the presence of squalene, while it has also shown antitumora effects against some types of cancer [49]. Reactive oxygen species and oxidative damage were observed to decrease, and protect human mammary epithelial cells (MCF10A) with hydroxytyrosol and squalene [21]. Mice fed an olive oil-based diet had a more beneficial effect than mice on a high corn diet, and, in addition, the tumours were less aggressive; the mechanisms of action are at the cellular level, involving changes in cellular membranes, signaling pathways, and gene expression, which led to less proliferation, more apoptosis, and less DNA damage [104].

A study on cultured human T-cell leukaemia cells demonstrated that δ-tocotrienol was the most potent growth suppressor, corresponding to G1 and G2/M cell cycle arrest and inter-nucleosomal DNA breakage associated with apoptosis, which is marked by activation of caspases-3, -6, -7, -9 and poly-ADP ribose polymerase (PARP). These effects are associated with the down-regulation of anti-apoptotic Bcl-2, Bcl-xL and X-IAP, and the down-regulation of nuclear factor-κB. Unlike mevalonate, farnesyl diphosphate, geranylgeraniol diphosphate, and cholesterol, the cytotoxic effects of δ-tocotrienol are blocked by squalene; however, the inhibition of cholesterol synthesis is not associated with a disruption of the function of squalene, leading to intracellular accumulation of squalene and promotion of apoptosis in leukaemia cells [81].

Human lymphoma and myeloma cell lines were subjected to a competitive proliferation experiment in which a subpopulation of cancer cells was found that is auxotrophic for cholesterol with a high dependence on cholesterol uptake. The deletion of squalene monooxygenase (SQLE) is the basis of cholesterol auxotrophy, notably in anaplastic lymphoma kinase (ALK) positive lymphoma cell lines and primary tumours [105]. Deficiency of squalene monooxygenase results in accumulation of intracellular squalene, which is an intermediate in cholesterol biosynthesis. In the present study, the authors demonstrate the importance of cholesterol uptake through the LDL receptor for the growth of cultured lymphoma cells and patient-derived xenografts [81,105].

Squalene was administered as a free compound and as an encapsulated PLGA nanoparticle, and in both forms exhibited low bioavailability and weak anticancer activity, with an IC_50_ of ~140 µg/mL against Caco-2 cells (human colon carcinoma cells), resulting from disruption of redox balance and induction of apoptosis [106]. Likewise, the inhibitory effect of the squalene-containing liposomal formulations on melanoma cell viability was observed at IC_50_ values of 225 µM and 215 µM in the B16-F10 and A-375 cell lines, respectively. In addition to direct cytotoxicity, recent studies have shown that squalene metabolism—through the action of squalene epoxidase- is involved in tumor immune evasion in the case of pancreatic cancer; thus, targeting squalene biosynthetic pathways could be an additional anticancer mechanism [107].

### 3.7. Sterols

#### 3.7.1. Chemical Composition of Sterols

The basic structure of sterols is derived from sterane, hydroxylated at C3, and consists of double bonds. The most important sterols found in olive oil include β-sitosterol, campesterol, stigmasterol, and Δ5-avenasterol [108].

#### 3.7.2. Health Benefits of Sterols and Anti-Cancer Properties

These are natural compounds found in the unsaponifiable fraction of vegetable oils. They differ from animal-derived oils in that the latter produce only cholesterol. Vegetable oils yield a diverse range of sterols that offer health benefits to humans—including lowering harmful (LDL) cholesterol, regulating the immune response, and reducing the risk of cardiovascular disease [108]. The composition of sterols varies depending on the olive cultivar. Olive oil contains several types of sterols; the most important are demethel sterol, monomethyl sterol, and dimethyl sterol, which are the most abundant in olive oil and are crucial for detecting adulteration during oil blending, as this is essential for authenticating olive oil [108].

Sterols have garnered increasing attention due to their potential anticancer properties. Studies indicate their ability to influence several pathways involved in tumor growth and development. Administration of β-sitosterol glycoside in mice was associated with increasing expression of Th1 cytokines, specifically IFN-γ and IL-2, compared to Th2 cytokines. This observation is explicitly linked to a predominant induction of the Th1 response, which aligns with the claim’s description of a Th1 shift [109]. Furthermore, they inhibit cancer cell proliferation by activating receptors such as LXRβ and LXRα. These receptors contribute to reduced estrogen receptor expression and increased p53 protein expression [110]. They also arrest the cell cycle at the G1 phase and modulate cholesterol metabolism, which helps reduce the division and growth of breast cancer cells [51].

Plant sterols, particularly β-sitosterol, may exert anticancer effects through the modulation of inflammatory and tumor-associated signaling pathways. In breast cancer, NF-κB inhibition has been associated with reduced production of IL-6, IL-8, VEGF, and MMP-9, mediators involved in tumor progression, invasion, and angiogenesis [51].

A Study has also shown their role in increasing the activity of glutathione peroxidase, superoxide dismutase (SOD), and catalase [111]. Furthermore, they prevent radiation-induced DNA damage and reduce oxidative stress, supporting their role as an antioxidant. They also reduce sphingomyelin and activate sphingomyelinase by increasing ceramide, which is considered a tumor-suppressing lipid [51].

### 3.8. Tocotrienol

#### 3.8.1. Chemical Composition of Tocotrienols

It is a compound from the vitamin E family, an unsaturated vitamin E analogue characterized by the presence of three double bonds in its side chain, and has four main types: α-tocotrienol, β-tocotrienol, γ-tocotrienol, and δ-tocotrienol [112].

#### 3.8.2. Health Benefit of Tocotrienols and Anti-Cancer Properties

Tocotrienols offer numerous benefits, acting as antioxidants and anti-inflammatories, reducing blood cholesterol levels, and possessing neuroprotective effects. They also combat chronic diseases [112]. Furthermore, their role extends to improving female reproductive health and metabolic syndromes, as well as protecting the liver and skin. They also contribute to cardiovascular health and bone density [113].

Tocotrienol induces cancer cell death through several mechanisms that alter mitochondrial function and modify protein balance. It increases pro-apoptotic proteins (Bax) and decreases anti-apoptotic proteins (Bcl-2), thus contributing to cancer cell death [114]. It showed effects on pancreatic, intestinal, and blood cancers by inhibiting cell growth and division and arresting the cell cycle. Furthermore, it inhibits tumor angiogenesis by reducing VEGFR, VEGF, and HIF-1α levels, thereby reducing blood and nutrient supply to the tumor site. It also reduces cancer cell migration through MAPK and AKT signaling pathways. In addition, it plays a significant anti-inflammatory role by inhibiting inflammatory pathways including NF-κB, which helps cancer cells survive [112]. Collectively, the biologically active compounds in olive oil exert their anti-cancer effects through distinct molecular pathways: cell cycle arrest, induction of apoptosis, suppression of angiogenesis, and metabolic disruption of cancer cells.

Table 2 and Figure 2 summarize these mechanisms along with the corresponding experimental models, cancer types, and outcomes.

**Table 2:** Anticancer mechanisms of olive oil bioactive compounds.

Name of Compounds	Anticancer Mechanism	In Vitro Evidence	Animal Evidence	Human Evidence	References
Oleuropein	Anti-proliferative, anti-metastatic, and anti-angiogenic effects, reduced tumour volume and skin carcinogenesis. Inhibition of NF-κB, COX2, CREB, and wnt/β-catenin pathways; blocking of MAPKs, ERK, and AKT; modulation of PPAR receptors and VEGF/MMP activity; chelation of copper and iron inducing pro-oxidant ROS activation	Breast, colorectal, prostate, thyroid, hepatoma, cervical, mesothelioma, osteosarcoma, and pancreatic cancer cell studies show anti-proliferative, pro-apoptotic, anti-migratory, and pathway-inhibitory effects; include 50–200 μg/mL in MCF-7, 80 μmol/L in PC-3, 150–200 μM in HeLa, and 22 μg/mL in mesothelioma fraction studies	Reduced colitis-associated colorectal cancer in mice and prevented UVB-induced skin carcinogenesis; 10 and 25 mg/kg oral oleuropein were used in the skin model	Limited clinical evidence	[19,20,30,52,55,61,115,116]
Oleocanthal	Induced apoptosis, anti-proliferative and anti-cancer cell growth effects, and induced programmed cell death. It has few toxic effects on normal cells. Upregulation of Bax, caspase-3, and caspase-9 with downregulation of Bcl-2. It inhibits cell growth in human breast cancer. It inhibits HGF-stimulated C-Met activity and cell division-inducing signaling pathways. It suppresses tumor growth in hepatocellular carcinoma by inhibiting epithelial–mesenchymal transformation (EMT), reducing Twist transcription factor expression, and contributing to reduced STATE 3 translocation to the nucleus and decreasing its DNA-binding activity, thereby lowering cyclin D1 levels	Breast cancer MCF-7, BT, 474, and MDA-MB 231, and TNBC cell studies show reduced proliferation and altered pathways; the 2023 TNBC study used varying concentrations for 48 h, and the 2019 oral breast cancer study validated anti-breast-cancer activity	In mice, 5 mg/kg reduced breast tumor growth by 60% and suppressed angiogenesis/mitosis	A pilot randomized trial in early-stage CLL used 40 mL/day high oleocanthal/oleacein EVOO and found favorable blood and apoptotic marker changes, but this is preliminary and not definitive cancer efficacy	[38,46,117,118]
Oleacein	Induced apoptosis, cell cycle blockade, and angiogenesis suppression. Cell cycle arrest via Cdk2 phosphorylation; upregulation of BAX with downregulation of Bcl-2 and MCL-1; inhibition of clonogenicity and HDACs; disruption of angiogenesis and metastatic signaling cascades. Increased cleaved cytokeratin-18 and Fas/Apo-1; decreased survivin and cyclin D	Multiple myeloma, colorectal, prostate, hepatocellular, pancreatic, and lung cancer cell lines. Clinical trial (early-stage CLL patients). SH-SY5Y neuroblastoma cells showed reduced proliferation, S-phase arrest, apoptosis, and reduced migration/adhesion	No clear animal anticancer study was retrieved	Human anticancer data are limited to the same pilot CLL EVOO trial, which tested oleocanthal/oleacein-rich oil rather than oleacein alone	[20,38,71,81,83,85]
Hydroxytyrosol	It has anti-cancer and anti-inflammatory properties. At a certain concentration, it has been shown to inhibit cell growth by 50%, contributing to chemoprevention by reducing cell proliferation. It increases the expression of genes that lead to programmed cell death by activating caspase enzymes, reduces the expression of genes that inhibit programmed cell death, and increases the level of reactive oxygen species.	Breast, colon, prostate, hepatocellular, pancreatic, and thyroid cancer cell lines showed growth inhibition, apoptosis, ROS-related effects, and pathway suppression; examples include 50 μg/mL in MCF-7 and 80 μmol/L in PC-3	In vivo animal assays support anti-proliferative and pro-apoptotic effects.	Human cancer-specific evidence is limited; one human blood-cell DNA damage study suggests chemo-preventive plausibility, not treatment benefit	[26,55,93,94,95,119]
Tyrosol	Suppressed inflammation, induced apoptosis, and anti-proliferative activity including imatinib-resistant cells	Breast, colorectal, prostate, liver cancer cell lines, K562 cell line. Pancreatic cancer cell work compared tyrosol with oleuropein/hydroxytyrosol and found weaker activity; other evidence mainly points to mechanistic/adjunctive interest	No strong animal anticancer evidence	No Human anticancer evidence	[20,26,31,100,101,120,121]
Tocotrienol	It activates programmed cell death pathways, thus inducing cancer cell death. It also prevents the formation of new blood vessels to nourish the tumor, in addition to regulating cellular signaling pathways and modulating estrogen-related mechanisms	Breast cancer, including MCF-7, MDA-MB-231 In vivo: mammary syngeneic model. Strong in vitro antiproliferative and apoptosis-inducing effects, often at low micromolar doses; examples include γ-tocotrienol at 4 μM and δ-tocotrienol at 10–20 μmol/L	Animal studies show tumor growth inhibition and dose effects, including 10 mg/mouse in one study and 3–100 mg/kg in others	Ovarian-cancer trial with median progression-free survival rising from 6.9 to 10.9 months, and another healthy subject study shows pharmacokinetic bioavailability only	[113,122,123,124,125]
Squalene	Induced cell cycle arrest and apoptosis; disrupted redox balance; reduced tumor immune evasion. Depletion of intracellular squalene activating caspases and downregulating Bcl-2/Bcl-xL/X-IAP; loss of squalene monooxygenase disrupting cholesterol synthesis; enhanced delivery via PLGA nanoparticles and liposomal formulations; modulation of squalene epoxidase (SQLE)	In vitro (T-cell leukaemia, lymphoma, myeloma, Caco-2, B16-F10, A-375 cell lines) and in vivo (pancreatic cancer model) The main support is adjunctive and formulation-related	Animal studies suggest tumor-growth inhibition/prevention, including 5% dietary squalene and 4.2 g/kg with ACNU in mouse models	No direct human anticancer evidence was retrieved here	[81,106,107,126,127,128]
Sterols	Strong inhibition of tumor growth, induction of apoptosis and angiogenesis, preventing metastasis. The apoptosis pathway is activated by increased caspase 9/3 expression, elevated BAX levels, and decreased levels of antiapoptotic proteins, thus promoting apoptosis, cell cycle arrest, Increased ROS in cancer cells	MCV-7 cell xenograft mice and HccLM3. β-sitosterol was reported active at 16 μM in prostate cancer cells	Preclinical studies support dose-dependent anticancer activity, and the review reports animal doses around 25–400 mg/kg	Limited clinical evidence exists	[129,130,131,132,133]

## 4. Doses and Concentrations Evaluated in Experimental and Human Studies

The doses and concentrations reported across in vitro, in vivo, and human studies are summarized in Table 3, providing an overview of the exposure levels used across different experimental settings.

### 4.1. Oleuropein

In vitro studies have shown that oleuropein exhibits broad anticancer effects through multiple mechanisms, including inhibition of proliferation, induction of apoptosis, cell-cycle arrest, and modulation of cancer-associated molecular pathways, with effects that are dose- and time-dependent. In breast cancer cells, delivering 110–200 µM oleuropein reduced cell viability and induced apoptosis [134]. Moreover, concentrations of approximately 100–400 µM inhibited proliferation, promoted apoptosis, and downregulated HIF-1α in colorectal cancer models [60]. In leukemia cells, 244 µM oleuropein significantly reduced K562 cell viability after 72 h in a dose- and time-dependent manner [63]. Neuroblastoma cells showed dose-dependent cytotoxicity at 350–400 µM [135], whereas in ovarian cancer models, low doses decreased cell viability and higher doses exerted anti-proliferative and pro-apoptotic effects [29]. Moreover, in vivo studies showed that oleuropein administered at 10–125 mg/kg or incorporated into the diet at 125 mg/kg reduced tumor growth, dysplasia, and metastasis [36,136].

Finally, a human study was conducted involving five middle-aged male and five middle-aged female volunteers, who each received two single doses of oleuropein—a low dose (51.1 mg) and a higher dose (76.6 mg)—as part of an investigation into the substance’s pharmacokinetics. No side effects or liver function abnormalities were observed during the trial. Furthermore, the plasma concentration of oleuropein was six times higher when administered in liquid form than in capsule form. The results also indicated that factors such as sex, enzymatic activity, and the form of the preparation influence the metabolism and absorption of oleuropein; additionally, differences in metabolism were noted between postmenopausal and premenopausal women. Moreover, oleuropein absorption and metabolism are influenced by multiple factors; further human studies are needed to better characterize its pharmacokinetic profile [19].

### 4.2. Hydroxytyrosol

Hydroxytyrosol has demonstrated anticancer activity at low-concentration dose ranges, even though higher concentrations have also been reported. In breast cancer models, hydroxytyrosol at 0.5–100 μM reduced mammosphere formation, migration, and invasion [137], while concentrations around 50 μg/mL reduced MCF-7 cell viability [55]. In prostate cancer cells, administration of 80 μM hydroxytyrosol increased apoptosis, superoxide production, autophagy defects, and mitochondrial dysfunction [93]. In colorectal cancer models, concentrations of 10–100 μM inhibited proliferation and reduced DNA damage [138], while nano-encapsulated hydroxytyrosol exhibited more potency than the delivery-free compound [139]. In vivo, hydroxytyrosol demonstrated antitumor effects at doses ranging from 0.5 to 250 mg/kg/day, including inhibition of mammary tumor growth at 0.5 mg/kg and suppression of tumor growth and AKT/NF-κB signaling in hepatocellular carcinoma models at 20 mg/kg [90,140].

Human evidence remains limited; reported is 25 mg/day for 12 months in a breast-cancer prevention study, which was associated with changes in breast density rather than anticancer activity [141]. Studies conducted in humans have shown that daily supplementation with hydroxytyrosol doses of 5–15 mg, either pure or in EVOO rich in polyphenols, over 3–26 weeks, improved oxidative stress with good tolerability and no potential short-term side effects. However, evidence regarding its long-term safety and the effects of high-dose exposure remains limited [87]. Based on the Nutrition and Allergy Committee, it was indicated that 5 mg of hydroxytyrosol and its derivatives should be consumed daily in olive oil to achieve its physiological effect, and its quantity in olive oil should not exceed 20 g; this is to protect low-density lipoprotein (LDL) molecules from oxidative damage and is not an anticancer dose recommendation [119].

### 4.3. Oleocanthal

Oleocanthal showed the strongest antiproliferative and cytotoxic activity among six olive oil secoiridoids in most of the 16 human cancer cell lines investigated after 72 h of treatment, with EC_50_ values generally ranging from 9 to 20 µM. In breast cancer cell lines, the EC_50_ values were 10.4 ± 0.8 µM in MDA-MB-231, 13.0 ± 0.6 µM in SK-BR-3, and 24.6 ± 2.6 µM in MCF-7 cells. Oleocanthal exhibited the strongest antiproliferative and cytotoxic activity, followed in descending order by oleuropein aglycone, ligstroside aglycone, oleacein, oleomissional, and oleocanthalic acid [142].

Injectable administration of oleocanthal at 5 mg/kg/day, three times per week, inhibited cancer-growth cells in a localized breast cancer implantation model in thymus-free mice. Researchers also found that oleocanthal at a concentration of 10 μM reduced the expression of phosphorylated mTOR in breast adenocarcinoma. Furthermore, other researchers investigated the effect of oleocanthal on human cancer cells, showing that treatment with 20 μM oleocanthal in the absence of serum resulted in a loss of cell adhesion within 30 min and 100% cancer cell death 24 h after treatment, confirming its role in inducing cancer cell death [71].

### 4.4. Oleacein

The doses of oleacein required to produce anticancer effects have been studied mostly in in vitro models. Multiple myeloma cell lines were inhibited for cell viability after 48 h of treatment with IC_50_ values between 5.0 and 20.0 µM, while peripheral blood mononuclear cells from healthy donors were not affected by the drug. This would indicate an in vitro selectivity of at least 15.0, which is a favorable index [81]. In melanoma cells (501Mel), a low micromolar IC_50_ dose of the compound was enough to arrest cells in the G1/S phase, induce DNA fragmentation, and downregulate antiapoptotic genes, such as Bcl-2, MCL1, and the protooncogene mTOR [82].

On the clinical side, a pilot randomized clinical trial was conducted in patients with early-stage chronic lymphocytic leukemia, and daily oleacein- and oleocanthal-enriched extra virgin olive oil resulted in a significant increase in apoptotic indicators and a reduction in survival markers, the first human-level study of the biological activity of the compound in a cancer setting [38]. One should, however, note that the concentrations needed for anticancer activity in vitro are significantly higher than are likely to be reached with dietary supplementation of extra virgin olive oil alone, which calls for standardization of the supplementation protocols and further clinical studies to determine optimal and safe dosing of oleacein for cancer prevention and treatment [20].

### 4.5. Tyrosol

The doses required for the anticancer activity of tyrosol and its derivatives are cancer-type and structure-dependent. The derivatives with isoxazole and triazole groups showed good antiproliferative activity against chronic myeloid leukemia (CML) K562 cells, with IC50 values of 45 µM and 61 µM, respectively, both of which exhibited significant activity against primary chronic myeloid leukemia (CML) cells resistant to imatinib [31]. Olive leaf extract’s tyrosol has been shown to induce apoptosis and inhibit colony formation in glioblastoma models at IC_50_ concentrations and was also found to be synergistic with the chemotherapy drug temozolomide, suggesting a possible role for tyrosol in combination cancer therapy [143]. It should be mentioned that tyrosol alone is relatively non-toxic, but chemical derivatization or combination strategies increase the potency [144]. Moreover, tyrosol concentrations required to achieve anticancer effects in vitro are generally in the micromolar range and may exceed those attainable through dietary intake. Therefore, strategies to enhance its bioavailability, such as developing optimized formulations or modifying its chemical structure, warrant further investigation before its therapeutic potential can be evaluated in clinical trials [20,144].

### 4.6. Squalene

The doses of squalene needed to achieve anticancer activity are much higher than those of polyphenols in olive oil and are very dependent on the delivery system used. However, in its free form, squalene is poorly bioavailable and exhibits low anticancer activity. Researchers investigated other delivery methods to improve the therapeutic potential of squalene [106]. Encapsulated in PLGA (polylactic-co-glycolic acid) nanoparticles, squalene has shown an IC_50_ of around 140 µg/mL, with the PLGA formulation showing anticancer activity through disruption of redox imbalance and induction of apoptosis in human colon carcinoma Caco-2 cells, thereby intensifying the activity of squalene [106]. Likewise, IC_50_ values of 225 µM and 215 µM were obtained for squalene in the melanoma cell lines B16-F10 and A375, respectively, and this effect was further enhanced when the squalene was used in combination with the peptide lunasin, indicating a synergistic effect between the two bioactive components [126].

In addition, it has direct cytotoxicity. Recent studies have shown that squalene metabolism through the enzyme squalene epoxidase may be involved in the process of immune evasion of tumors in pancreatic cancer, and the targeting of the tumor suppressive function of squalene biosynthetic pathways at physiologically relevant concentrations may be considered as another anticancer mechanism that does not require high dosage [107]. However, the squalene content in EVOO (ranging from 200 to 7500 mg/kg) is higher than in other vegetable oils, but it may not be present in sufficient concentration to produce direct and effective anticancer effects in experimental models [49]. Consequently, there is a need to develop standardized squalene formulations and conduct further clinical studies to bridge the gap between promising experimental data and actual use in cancer prevention and treatment.

### 4.7. Sterol

The evidence supports that 1–2 g/day is an established dose range for LDL-cholesterol reduction [145], whereas the dose required to achieve anticancer effects in humans remains undetermined. At the same time, phytosterols and specific compounds such as β-sitosterol and stigmasterol exhibit broad anticancer activity in vitro and in animal models, including induction of apoptosis and suppression of tumor growth, invasion, angiogenesis, and metastasis [146,147,148]. Therefore, further clinical studies focusing on dose determination, efficacy, and safety are needed, as promising preclinical findings have yet to establish a safe and effective therapeutic dose for cancer treatment [149].

**Table 3:** Doses and Concentrations of Olive Oil Bioactive Compounds Evaluated in Experimental and Human Studies.

Name of Compounds	Level of Evidence	Dose and Concentration	Modele	Anticancer Mechanism	References
Oleuropein	In vitro	100–200 µM/ 200 µg/mL	MCF-7 human breast cancer cells	Reduced cell viability and proliferation, induced apoptosis, and caused cell-cycle arrest at the G0/G1 phase, inhibiting the G1-to-S transition, increased p21, and inhibited NF-κB and its oncogenic target cyclin D1, promoting cell death. Associated with activation of autophagy and modulation of LC3-II/LC3-I, Beclin-1, and p62. Suppressed PI3K/AKT signaling through reduced phosphorylation.	[29,32,55,134,150]
Oleuropein	In vitro	100 µM	Human mononuclear cells	Reduced γ-radiation-induced apoptosis, DNA damage, and micronucleus formation, while increasing cell viability and radiation tolerance.	[42]
Oleuropein	In vitro	IC_50_ 247.4–475.0 µM (MG-63); 359.9–798.7 µM (Saos-2)	Human osteosarcoma MG-63 and Saos-2 cells	Time- and concentration-dependent cytotoxicity and inhibition of cell proliferation.	[151]
Oleuropein	In vitro	IC_50_ = 59.96 µM	A549 human lung carcinoma cells	Inhibited proliferation and migration, reduced ROS/MDA, and induced G1 cell-cycle arrest.	[152]
Oleuropein	In vitro	IC_50_ = 400 and 312 µg/mL (MCF-7), and 700 and 275 µg/mL (MDA-MB-231)	MCF-7 and MDA-MB-231	Reduced viability, induced apoptosis through modulation of miRNAs and apoptotic/tumor- suppressor pathways: ↑ miR-125b, miR-16, miR-34a, p53, p21, TNFRSF10B, ↓ Bcl-2, Mcl-1, miR-221, miR-29a, miR-21.	[41]
Oleuropein	In vitro	250 µM (non-toxic; antiproliferative); 500 µM (apoptosis-inducing).	A375/BRAF melanoma cells, including PLX4032-resistant cells	Induced apoptosis and inhibited proliferation through downregulation of the pAKT/pS6 pathway; potentiated DTIC and particularly RAD001 cytotoxicity.	[43]
Oleuropein	In vitro	IC_50_, 48 h 25–500 µM	MDA-MB-231 and MDA-MB-468 TNBC cells.	Reduced proliferation and modulated genes/pathways related to apoptosis, cell death, stress response, mitotic cell cycle, and cancer progression.	[153]
Oleuropein	In vitro	25–400 µM	A2780S (cisplatin-sensitive) and A2780/CP (cisplatin-resistant) ovarian cancer cells	Reduced viability and cisplatin resistance in A2780S and A2780/CP ovarian cancer cells; promoted apoptosis through modulation of miR-34a, miR-125b, miR-16 and miR-21, with increased p21/p53/TNFRSF10B and decreased Bcl-2/Mcl1.	[33]
Oleuropein	In vitro	IC50 150.1 µM	MIA PaCa-2, BxPC-3, CFPAC-1 pancreatic cancer cells; HPDE non-tumorigenic pancreatic cells	Showed selective cytotoxicity toward MIA PaCa-2 pancreatic cancer cells while protecting non-tumorigenic HPDE cells; induced apoptosis through modulation of Bcl-2 family proteins and caspase-3/7 activation, with c-Jun/c-Fos suggested as potential mediators.	[100]
Oleuropein	In vitro	IC50 140 µM for SEM-1 and 50 µM for TCAM-2	TCAM-2 (intragonadal seminoma) and SEM-1 (extragonadal seminoma)	Reduced viability and proliferation, induced apoptosis through BAX upregulation, and inhibited migration/motility. Effects were associated with NF-κB inhibition, cyclin D1 downregulation, p21 upregulation, and reduced TGF-β1 expression.	[44]
Oleuropein	In vitro	100–500 µM	HepG2 hepatocellular carcinoma cells cultured as 3D tumor spheroids	Dose-dependently inhibited cell proliferation and tumor-spheroid growth. After 72 h, spheroid area was reduced by 63.56–88.06%.	[34]
Oleuropein	In vitro + in vivo	1.17 mM 100 µg/day subcutaneous injection/5 days	Normal human fibroblasts and tumor cell lines derived from advanced-grade human tumors (TF-1a; 786-O, T-47D, RPMI-7951, and LoVo)/rat	Disrupted actin filaments, inhibited tumor-cell proliferation, migration and invasiveness, and induced irreversible rounding of cancer cells. Oral administration produced complete regression of spontaneous tumors in mice within 9–12 days.	[62]
Oleuropein	In vivo	10 and 25 mg/kg, orally, twice daily for 30 weeks	Male hairless mice exposed to chronic UVB irradiation (36–180 mJ/cm^2^, 3 times/week for 30 weeks)	Prevented UVB-induced skin carcinogenesis and tumor growth. Reduced COX-2, VEGF, MMP-2, MMP-9, and MMP-13 expression and decreased Ki-67 and CD31-positive cells, suggesting inhibition of inflammation, angiogenesis, tumor-cell proliferation, and extracellular-matrix remodeling.	[30]
Oleuropein	In vivo	125 mg/kg	Ovariectomized nude mice bearing MCF-7 breast cancer xenografts	Potential anti-tumor and anti-metastatic activity.	[36]
Oleuropein	In vivo/cardioprotective/supportive evidence	100 and 200 mg/kg total, i.p., administered over 14 days	Rats with chronic doxorubicin-induced cardiomyopathy; DXR: 18 mg/kg i.p., divided into 6 doses over 2 weeks	Cardioprotective effect during chronic DXR treatment. Activated AMPK and Akt, suppressed iNOS, and helped restore eNOS balance, reducing nitro-oxidative stress, inflammation, and cardiac structural/functional damage.	[48]
Oleuropein	In vivo	20 mg/kg/day	Murine embryonic stem cells (aneuploid ESCs), with teratoma mouse model and metastatic teratomas	Inhibited metastasis, proteasome activator, counteracting the reduced proteasome activity associated with aneuploidy and helping suppress the metastatic phenotype.	[154]
Oleuropein	In vivo/protective/toxicity study	150 mg/kg/week	Female rats receiving cyclophosphamide (16 mg/kg/week) + epirubicin (2.5 mg/kg/week) for four cycles	Reduced antineoplastic-induced toxicity, including DNA damage, serum TNF-α and IL-6, and MDA levels; it increased GSH, SOD, and CAT in heart, kidney, and liver tissues.	[155]
Hydroxytyrosol	In vitro	30–50 µg/mL	MCF-7 human breast cancer cells	Reduced cell viability and proliferation, induced apoptosis, and caused cell-cycle arrest at the G0/G1 phase, inhibiting the G1-to-S transition, associated with ROS generation and PI3K/AKT inhibition.	[32,55]
Hydroxytyrosol	In vitro	50 µg/M	MCF-7 and T47D ER-positive breast cancer cells	More potent than oleuropein in reducing cell viability; suppressed migration/invasion through activation of autophagy-related pathways.	[150]
Hydroxytyrosol	In vitro	IC_50_ at 24 h: 27.3 µg/mL (Jurkat), 109.8 µg/mL (HL60); 45.7 µg/mL in Raw264.7	Human leukemia T cells: Jurkat and HL60; murine macrophages: Raw264.7	Reduced proliferation and induced apoptosis; caused G0/G1 cell-cycle arrest with reduced S phase. Inhibited PI3K signaling, with consequent activation of MAPK-related signaling. In Raw264.7 cells, reduced LPS-induced NO production, indicating an anti-inflammatory effect.	[156]
Hydroxytyrosol	In vitro	IC50 75.1 µM	MIA PaCa-2, BxPC-3, CFPAC-1 and HPDE cells	Showed cytotoxicity, induced apoptosis associated with changes in Bcl-2 family proteins and caspase-3/7 activation; c-Jun/c-Fos were suggested as potential mediators.	[100]
Hydroxytyrosol	In vitro	50–100 ppm	C32 melanoma and HEMa non-tumorigenic 3D spheroids	Reduced spheroid growth and cell viability, induced cell-cycle arrest and apoptosis, with relatively limited cytotoxicity in HEMa spheroids. Proteomic analysis showed downregulation of ERBB2, ERBB3, ERBB4, VEGFR-2 and WIF-1, with modulation of PI3K-Akt and MAPK/ERK signaling pathways.	[91]
Hydroxytyrosol	In vivo (toxicological/safety study)	NOAEL: 500 mg/kg/day	Rats	No adverse effects were observed at doses up to the proposed NOAEL. Potential supplement or preservative.	[157]
Hydroxytyrosol	In vivo	100 g/day	Adult Wistar rats model of glioma	Inhibited tumor growth and reduced lipid and protein oxidation while modulating enzymatic and non-enzymatic antioxidant defenses.	[35]
Hydroxytyrosol	Human–safety/clinical evidence	45 mg/day for 8 weeks	14 human volunteers with mild hyperlipidemia	No adverse effects on cardiovascular, lipid, inflammatory, liver, kidney, hematological, or electrolyte parameters. HT increased serum vitamin C approximately two-fold.	[39]
Oleocanthal (−)	In vitro	IC_50_: 4–20 µM	MCF-7, MDA-MB-231 and PC-3 human breast/prostate cancer cells; endothelial colony-forming cells	Inhibited cancer-cell proliferation, migration, and invasion and reduced the angiogenic marker CD31. Molecular studies showed inhibition of c-Met phosphorylation, supporting modulation of the HGF/c-Met signaling pathway. Anti-angiogenic activity in endothelial colony-forming cells	[158]
Oleocanthal	In vitro	IC_50_, 48 h IC_50_, 48 h 25–500 µM	MDA-MB-231 and MDA-MB-468 TNBC cells	Reduced proliferation and altered apoptosis, cell death, stress-response, and cell-cycle pathways; slightly stronger effect than oleuropein.	[153]
Oleocanthal	In vitro	3 µg/mL	MCF-7 and MDA-MB-231	Produced ROS, inhibited proliferation, promoted apoptosis, and induced cell-cycle arrest (G1 in MCF-7; S phase in MDA-MB-231), associated with reduced Cyclin D and inhibition of PI3K/AKT signaling.	[32]
Oleocanthal	In vitro	20 µM	Human malignant melanoma cells	Selectively inhibited melanoma-cell proliferation, associated with reduced ERK1/2 and AKT phosphorylation and decreased Bcl-2 expression	[159]
Oleocanthal	In vitro + in vivo	40 ng/mL 5 mg/kg	MDA-MB-231, MCF-7 and BT-474 breast cancer cells; MCF10A normal breast epithelial cells; orthotopic breast cancer model in athymic nude mice	Inhibited breast cancer cell growth. Dose-dependently reduced HGF-induced migration, invasion, and G1/S cell-cycle progression. Effects were associated with inhibition of HGF-induced c-Met activation and downstream mitogenic signaling, suppression of EMT and cellular motility. Suppressed tumor growth in the orthotopic mouse model.	[46]
Oleocanthal	In vitro + in vivo	5–10 µM; 5 mg/kg, i.p., 3×/week in mice	ZR-75-1 (luminal A) and MDA-MB-231 (TNBC); orthotopic ZR-75-1 xenograft and MDA-MB-231-Luc metastasis models	Markedly reduced migration and invasion. Suppressed tumor progression and metastatic colonization; modulation of the SMYD2–EZH2–STAT3 signaling pathway	[160]
Oleocanthal (−)	In vitro + in vivo	20 μM 5 mg/kg	Various cancer cell lines; genetically engineered mice developing pancreatic neuroendocrine tumors	Induced lysosomal membrane permeabilization (LMP) and lysosomal damage, resulting in cancer-cell toxicity. In vivo, oleocanthal reduced tumor burden and prolonged mouse survival.	[79]
Oleocanthal (−)	In vitro + in vivo	30–50 µM 5 mg/kg/d or 10 mg/kg/d, i.p.	Human hepatocellular carcinoma (HCC) cells; orthotopic HCC mouse model; in vivo lung metastasis model	Inhibited proliferation, apoptosis, migration, invasion, tumor growth and metastasis, associated with suppression of the JAK1/2–STAT3 pathway, Twist-mediated EMT, Cyclin D1, Bcl-2, survivin and MMP-2	[76]
Oleocanthal	In vivo/pharmacokinetic	5 mg/kg, i.p.	Mice	Rapidly metabolized/disappeared from plasma; 13 metabolites identified, with oleocanthalic acid and tyrosol sulfate proposed as potential biomarkers of exposure.	[25]
Oleacein	In vitro	IC50 5.0–20.0 µM	Multiple myeloma primary samples and cell lines/501Mel human cutaneous melanoma cells	Reduced viability and clonogenicity, induced apoptosis, and inhibited HDACs, with Caspase-8/Sp1 involvement; enhanced the anti-MM activity of carfilzomib synergistically Induced G1/S arrest and DNA fragmentation, with modulation of apoptosis- and proliferation-related genes and miRNAs.	[84,85]
Oleacein	In vitro	12.5 µM	RAW 264.7 macrophages; PLA_2_/5-LOX enzyme systems	Reduced L-citrulline and NO production in LPS-stimulated macrophages and inhibited PLA_2_/5-LOX activity, indicating modulation of NO- and arachidonic-acid-dependent inflammatory pathways.	[161]
Oleacein	In vivo/pharmacokinetic	0.3 mg/mL	Rats; plasma and tissues collected at 1, 2 and 4.5 h	Unmetabolized, detected in plasma, stomach, small intestine, liver, and especially heart. Nine metabolites were identified, including five conjugated metabolites.	[162]
High OC/OL-EVOO (oleocanthal + oleacein)	Human–pilot clinical evidence	40 mL/day for 3 months (DI1): EVOO containing 416 mg/kg OC + 284 mg/kg OL; 40 mL/day for 6 months (DI2) with high OC/OL-EVOO	20 patients with untreated CLL, Rai stages 0–II in DI1; 22 patients in DI2	Favorable changes in hematological and apoptotic markers. DI2 showed decreased white blood cell and lymphocyte counts, increased ccK18, Apo1-Fas and p21, and decreased Survivin and Cyclin D, consistent with increased apoptosis and reduced proliferative signaling.	[38]
Tyrosol	In vivo	10 mg/kg/day, i.p.	Colorectal cancer mouse models	Showed antitumor activity and selective effects against CRC cells, associated with reduced ROS and inflammatory cytokines and inhibition of HIF-1α/NF-κB signaling.	[37]
Squalene	In vitro selective cytoprotective evidence	12.5–25 µM	Normal human bone-marrow-derived colony-forming units (CFUs); neuroblastoma cell lines, including 5 MYCN-amplified lines	selectively protected normal bone-marrow-derived CFUs from cisplatin-induced toxicity and apoptosis without protecting neuroblastoma cells from chemotherapy-induced cytotoxicity.	[163]
β-Sitosterol	In vitro	Physiological concentration range	MCF-7 and multidrug-resistant NCI/ADR-RES cells	Reduced cancer-cell viability, induced G0/G1 arrest in MCF-7 cells, and inhibited ABCB1/P-glycoprotein activity.	[146]
Pinoresinol-rich EVOO	In vitro	200 nM	Colorectal cancer cells	Reduced colorectal cancer cell viability and induced apoptosis and G2/M arrest, particularly in p53-proficient cells, through activation of the ATM–p53 pathway and downregulation of cyclin B/cdc2.	[147]
Campesterol, stigmasterol and β-sitosterol	Human	Highest tertile of pre-diagnosis dietary intake	Ovarian cancer patients	Higher intake was associated with better overall survival, with HRs of 0.54, 0.60 and 0.63, respectively.	[148]

## 5. Potential Combination Effect of Olive Oil Phytochemicals and Conventional Treatments

Increasing evidence suggests that olive oil phytochemicals may improve the therapeutic efficacy of conventional anticancer treatments through complementary molecular mechanisms. Furthermore, epidemiological evidence suggests that olive oil consumption is associated with health benefits and may provide a basis for developing novel cancer prevention strategies. Olive oil phytochemicals have shown synergistic or additive effects with conventional treatments in preclinical studies, but direct clinical evidence is still sparse. The strongest support is for oleuropein, hydroxytyrosol, and oleocanthal-rich olive oil in combination with standard drugs or drug-like agents. Some combinations are additive or even antagonistic depending on dose and ratio.

Oleuropein is a major active component. In vitro studies show that oleuropein has anti-tumor effects and changes microRNA (miRNA) patterns differently than the total Oleaeuropaea (olive) leaf extract. Combining oleuropein with the chemotherapy drug temozolomide (TMZ) increased the drug’s therapeutic effect by up to 2.83 times. mRNA expression levels of p53, a senescence factor, interleukin (IL)-6, matrix metalloproteinases (MMPs), and nuclear factor kappa B1 (NF-κB1), as senescence and senescence-associated secretory phenotype SASP factors, and Bcl-2 and Bax as senolytic markers were increased by temozolomide (TMZ) in glioblastoma (GB) cell lines T98G and U87MG. In addition, the *Oleaeuropaea* L. leaf extract OLE + TMZ reduced the proportion of senescent cells (*p* < 0.001) and their capability to form colonies and increased more than six times in the Bax/Bcl-2 ratio compared to TMZ alone, which induced apoptosis-like morphological features [164].

The synergistic effects of oleuropein and azacitidine suggest a potential therapeutic strategy for acute myeloid leukemia AML by targeting epigenetic mechanisms and miRNA pathways. Despite conventional treatments, significant challenges remain. Epigenetic alterations, particularly dysregulated DNA methylation and microRNA (miRNA) expression, are implicated. Both azacitidine and oleuropein reduced cell viability and induced apoptosis in a dose- and time-dependent manner. Notably, the combination treatment significantly enhanced apoptosis with a 2.5-fold increase in HL-60 cells. Furthermore, the treatment modulated miRNA expression, upregulating miR-149-3p and miR-375 while downregulating miR-574-5p [165].

Another study demonstrated that a high dose of oleuropein triggers cell apoptosis in A375 human melanoma cells, whereas a non-toxic dose of 250 µM inhibited cell proliferation and suppressed the pAKT/pS6 signaling pathway. A dose of 250 µM Ole did not potentiate the antitumor activity of the BRAF inhibitor Vemurafenib (PLX4032), but it significantly increased the cytotoxic effect of Dacarbazine (DTIC). The major synergistic effect was also observed in the association between oleuropein and Everolimus (RAD001), including in PLX4032-resistant BRAF melanoma cells, which possibly cooperate in the inhibition of the pAKT/pS6 pathway. Furthermore, an olive leaf extract enriched in oleuropein exhibited more effective and able to additional improve DTIC and RAD001 efficacy on BRAF melanoma cells with respect to oleuropein alone. Therefore, oleuropein represents a natural product able to potentiate a wide array of chemotherapeutics against BRAF melanoma cells affecting the pAKT/pS6 pathway [43]. Oleuropein alone exhibits remarkable anti-proliferative and anti-migratory activity against cancer cells. Co-administration with 2-methoxyestradiol significantly improves therapeutic efficiency. Both anti-migratory and anti-proliferative effects of oleuropein were demonstrated on human osteosarcoma OS cells. Anticancer effects of oleuropein were significantly enhanced after 2-ME addition. Treatment of 143B OS cells with oleuropein, alone or in combination with 2-ME, resulted in induction of autophagy [166].

Adriamycin (ADR) is an anthracycline antibiotic widely used as a chemotherapeutic agent, although it presents significant side effects. In co-treatment, oleuropein with Adriamycin ADR (50 nM) was used in MG-63 human osteosarcoma cells. The observed alterations in gene, protein, and metabolite levels denote that oleuropein not only inhibits MG-63 cell proliferation and potentiates ADR’s cytotoxicity, but also exerts its action, at least in part, through the induction of autophagy [167].

Doxorubicin (Dox) is a highly effective chemotherapeutic agent active in the treatment of hematological and solid tumors. The effective use of Dox in cancer therapy has been limited due to its well-known cardiotoxic side effects, mainly mediated by oxidative damage. Hydroxytyrosol (HT)—the primary phenol found in olive oil—protects heart muscle cells from damage caused by Dox without reducing the cancer-killing potency of doxorubicin on osteosarcoma cells. It achieves this by reducing harmful ROS, regulating SOD2 levels, and balancing apoptotic markers like Bcl-2/Bax. In a previous study, the effect of HT was tested on Dox-induced cardiotoxicity by using a combination of biochemical and cellular biology techniques. Interestingly, HT was able to counteract Dox-induced cytotoxicity in cardiomyocytes by acting on the SOD2 level and the oxidative response, as well as on apoptotic mechanisms mediated by Bcl-2/Bax. At the same time, HT did not interfere with the antitumorigenic properties of Dox in osteosarcoma cells. This study identifies new, beneficial properties for HT and suggests that it might be a promising molecule for the development of additional therapeutic approaches aimed at preventing anthracycline-related cardiotoxicity and improving long-term outcomes in antineoplastic treatments [168]. A study showed the promising synergistic efficacy of DOX with oleuropein against breast tumor xenografts in MDA-MB-231 human breast cancer cells. A sharp drop, to 48.7 mm^3^, in the volume of the tumor was observed with combined treatment, a more than 3-fold decrease. The effect was mediated through the induction of apoptosis via the mitochondrial pathway and the downregulation of the pro-proliferation proteins nuclear factor-kappa B, and cyclin D1. Furthermore, it inhibited the expression of Bcl-2 and survivin. This inhibition could explain the cooperative suppression of the proliferation of breast tumor xenografts and the induction of apoptosis by the combined effect of the compounds used [169]. To enhance anticancer potency, the combination chemotherapy of HT-29 cells with biodegradable, pH-sensitive nanoparticles for co-delivery of hydroxytyrosol and doxorubicin was used, with co-delivery of doxorubicin and hydroxytyrosol resulting in greater apoptosis in HT-29 cells compared with single drugs, and reports significant downregulation of hTERT, CREB1, and CREB2 [98].

The first report investigated the potential role of miR-34a, miR-16, miR-21, and miR-125b in the regulation of genes and signaling pathways acquired drug resistance by oleuropein and/or cisplatin in ovarian cancer cells, which potentially resulted in apoptosis induction, cell proliferation inhibition, and cisplatin resistance decline in A2780/CP and A2780S ovarian cancer cells. Further, upon combination treatment with oleuropein, the expression of P21, P53, and TNFRSF10B increased, while that of Bcl-2 and Mcl1 decreased [33].

Hydroxytyrosol is wide use in combined therapies with paclitaxel against breast cancer, as adjuvant and sensitization therapy. Combination with hydroxytyrosol would also guarantee a less oxidative effect of chemotherapeutic drugs that could potentially improve patient wellness and significantly reduce tumor volume when compared with paclitaxel alone in MCF-7 and MDA-MB-231 breast cancer cell lines in vitro and in vivo experiments. Additionally, the combination improved the antioxidant status without compromising the antitumor activity of conventional chemotherapy [97]. Hydroxytyrosol also enhances cetuximab’s inhibitory effects on HT-29 and WiDr colon cancer cell growth at concentrations 10 times lower than when used as single agents, reflecting synergistic preclinical activity. This reduction was linked to cell cycle blockade, happening at G2/M phase by reducing levels of specific cyclins B, D1, and E, and cyclin-dependent kinase (CDK) 2, CDK4, and CDK6 down-regulation, and to the associated overexpression of CDK inhibitors p21 and p27. HT and cetuximab stimulated a caspase-independent cell death cascade, promoted translocation of apoptosis-inducing factor (AIF) from mitochondria to the nucleus, and activated the autophagy process. Moreover, containing olive oil in the diet during cetuximab chemotherapy of colon tumors may be enhancing the effects of epidermal growth factor EGFR inhibitors [170].

Oleacein demonstrated anticancer activity against several tumors such as melanoma. Oleacein induced cell growth inhibition in melanoma cells, G1/Sphase arrest, DNA fragmentation, and downregulation of genes encoding antiapoptotic (Bcl-2 and MCL1) and proproliferative (c-KIT, K-RAS, PIK3R3, mTOR) proteins, whereas it increased transcription levels of the proapoptotic protein BAX. These results demonstrate the ability of Oleacein to be used as an anticancer and chemopreventive agent for cancer therapy [85].

Furthermore, a previous study evaluated the ability of low doses of extra-virgin olive oil extract (EVOOE) to modulate the in vitro activity of paclitaxel or mitomycin, two antineoplastic drugs used in the management of different types of cancer. EVOOE significantly inhibited the proliferation and clonogenic ability of T24 and 5637 bladder cancer cells in a dose-dependent manner. Furthermore, EVOOE treatment showed a marked growth arrest before mitosis in the G2/M phase for both cell lines. In addition, simultaneous treatment with mitomycin C and EVOOE reduced drug cytotoxicity by inhibiting ROS production. Conversely, cotreatment of T24 cells with paclitaxel and the polyphenol extract strongly increased apoptotic cell death compared to paclitaxel alone [171].

The best-supported combination is multi-target additivity: olive oil phenolics can amplify anti-inflammatory, anti-angiogenic, antimicrobial, and anticancer pathways that conventional treatments aim to modulate, making those promising adjuvants or sensitizers rather than replacements. For oleocanthal, oleacein, and tyrosol, the evidence base is still needed before true treatment synergy can be demanded.

## 6. Commercial EVOO-Based Products and Their Functional Benefits

Numerous commercially available EVOO-based products have explored the potential of phenolic-rich olive oil in cancer-related and supportive health care. The Governor^®^ and Atsas^®^, naturally oleocanthal-rich EVOOs from Greece and Cyprus, respectively, showed greater cytotoxic activity against prostate and breast cancer cells than oleocanthal-poor oils, with activity correlated with oleocanthal content and associated with lysosomal membrane permeabilization in preclinical models [79]. The Governor^®^ was also investigated in a pilot randomized dietary intervention using high-oleocanthal/oleacein EVOO in early-stage chronic lymphocytic leukemia patients (40 mL/day; 416 mg/kg oleocanthal and 284 mg/kg oleacein), where consumption was associated with favorable changes in hematological, apoptotic, and cell-proliferation markers, including increased apoptotic markers and decreased Survivin and Cyclin D levels. However, the study was not considered to study anticancer efficacy [38]. The same commercial EVOO has also been evaluated in a randomized controlled trial in individuals with mild cognitive impairment, in which the intervention provided 30 mL/day of EVOO containing approximately 1200 mg/kg total polyphenols, predominantly oleocanthal and oleacein [172]. PAMAKO^®^, a commercially available high-phenolic EVOO from Greece, has also been investigated as a source of bioactive compounds for nutraceutical development. In a recent preclinical study, phenolics extracted from PAMAKO^®^ were concentrated into an experimental formulation containing 40% (*w*/*w*) total phenolics, which demonstrated tumor-suppressive activity in a triple-negative breast cancer xenograft model [173]. In addition, Bi1 Procare^®^ (Adventia Pharma) is a commercial oral immunonutrition formula containing high-calorie, high-protein oral nutritional supplements, EVOO, fiber, arginine, omega-3 fatty acids (EPA and DHA), and nucleotides. In a randomized multicenter trial involving patients undergoing surgery for gastric, esophageal, and biliopancreatic tumors, it was associated with fewer interventions and improved postoperative cellular health [174]. More recently, Corbella^®^ EVOO, a commercially available EVOO based on the native Corbella olive variety, was evaluated in a randomized crossover trial in healthy adults. Four weeks of consumption markedly increased urinary hydroxytyrosol- and tyrosol-derived metabolites compared with regular olive oil and was accompanied by exploratory improvements in waist-related measures and HbA1c [175]. Although these findings are not cancer-specific, they provide human evidence that a commercially available phenolic-rich EVOO can increase exposure to bioactive olive-oil phenolics and may utilize broader metabolic effects. Collectively, these examples illustrate the expanding use of commercial EVOO products as sources of rich bioactive phenolics and as components of functional or supportive nutritional strategies, while emphasizing that direct evidence for cancer prevention or therapeutic efficacy of intact commercial EVOOs remains undetermined. Further evidence underscores the importance of product-specific phenolic characterization: a randomized clinical trial found that higher-phenolic EVOO consumed at a lower daily dose yielded greater health benefits than lower-phenolic EVOO consumed at a higher dose, despite similar daily phenolic intake. These findings emphasize the value of quantifying phenolic content when evaluating commercial EVOO products and their potential health benefits [176].

## 7. Conclusions

Cancer is still a major health problem worldwide, and finding ways to prevent cancer in a safer and more accessible way is still a priority. Among the distinct bioactive polyphenolic components of EVOO, oleuropein, hydroxytyrosol, oleocanthal, and oleacein have demonstrated broad and potentially relevant anticancer activities across different experimental cancer models.

One of the most promising anticancer effects of olive oil bioactive compounds is their ability to inhibit cancer cell proliferation and induce apoptosis. However, their potency and mechanisms vary according to the compound and tumor model. For example, oleacein inhibited the viability of human 501Mel melanoma cells in a time- and concentration-dependent manner, with IC_50_ values of 81.9 ± 6.9 μM after 48 h and 19.1 ± 5.8 μM after 72 h, accompanied by G1/S cell-cycle arrest and molecular changes consistent with apoptosis [85]. Hydroxytyrosol also induced apoptotic cell death and mitochondrial dysfunction in human colorectal cancer cells through ROS generation, whereas the nonmalignant colon epithelial cells were less affected under the tested conditions [93]. These findings suggest that redox-dependent mechanisms may contribute to cancer-cell inhibition in selected experimental systems. Evidence from in vivo studies further supports the potential relevance of these effects; dietary oleuropein reduced tumor incidence in the medial colon [136]. Also, dietary δ- and γ-tocopherol reduced mammary tumor burden in rats and colon cancer cell models. Notably, these tocopherols did not prevent tumorigenesis in the separate HER2/neu-driven breast cancer model [177]. In MDA-MB-231 breast cancer cells, β-sitosterol reduced cell growth by 66% after three days and 80% after five days, while increasing apoptosis sixfold compared with cholesterol-treated cells [178].

Olive oil bioactive compounds may also stimulate several interconnected pathways involved in cancer development, including inflammation, oxidative stress, cellular survival, and metabolic signaling. In a mouse model of colorectal cancer, oleuropein reduced colonic tumor growth and decreased intestinal concentrations of IL-6, IFN-γ, TNF-α, and IL-17A [89,101]. Modifications in NF-κB, Wnt/β-catenin, PI3K/AKT, and STAT3 signaling were also reported, supporting a potential relationship between inflammatory regulation and colorectal tumor development [32,58,61]. Oleocanthal provides complementary evidence involving STAT3 and c-Met signaling. In hepatocellular carcinoma models, treatment with 5 to 10 mg/kg/day of oleocanthal reduced tumor progression in orthotopic models and inhibited metastatic development in lung-metastasis models. The reported molecular effects included suppression of STAT3 activation and downstream mediators involved in tumor survival, proliferation, and invasion. These findings support further investigation of c-Met/STAT3 signaling as a potential therapeutic target, although the contribution of individual downstream pathways requires additional validation. Moreover, the effects on redox balance and signaling are not uniform across compounds or experimental systems. Although Hydroxytyrosol can act as an antioxidant, its reported effects in colorectal cancer cells involve ROS accumulation, mitochondrial dysfunction, and apoptosis [75,83,158]. On the contrary, tyrosol has demonstrated protective activity against oxidative injury in Caco-2 intestinal cells exposed to oxidized low-density lipoprotein [106]. Pretreatment with tyrosol reduced cellular injury and membrane damage, supporting cytoprotective activity under oxidative stress, but does not establish direct anticancer efficacy.

Intervention in cancer spreading and tumor vascularization represents another promising direction, particularly because tumor growth alone does not begin to affect metastatic progression. Oleocanthal has demonstrated activity in models addressing both primary tumor growth and metastasis. In addition to its effects in hepatocellular carcinoma models, an orthotopic human MDA-MB-231 breast cancer xenograft study reported reduced tumor growth following oleocanthal administration, together with reduced expression of c-Met, the proliferation marker Ki-67, and the vascular marker CD31 [30,65]. These observations support further investigation of its effects on tumor-associated angiogenesis and growth factor signaling. Further evidence suggests that angiogenesis may be a shared biological target of oleocanthal and oleacein. In vitro endothelial cell experiments and complementary angiogenesis models showed that both compounds inhibited endothelial proliferation, invasion, and tube formation. Oleacein additionally reduced endothelial-cell migration and induced apoptosis [86,158]. These results provide mechanistic evidence of anti-angiogenic activity, but they do not independently demonstrate suppression of tumor vascularization in different cancer types.

Modulation of estrogen signaling and enhancement of treatment response establish particularly promising ways in hormone-responsive breast cancer. Oleocanthal inhibited the growth of BT-474, MCF-7, and T-47D breast cancer cells and acted synergistically with tamoxifen. In an orthotopic xenograft model, oleocanthal treatment inhibited 97% of tumor growth relative to controls. The study also reported a reduction in estrogen receptor levels. Another piece of evidence indicates that in an estrogen-stimulated MCF-7 xenograft model [56], dietary supplementation with α-, γ-, or δ-tocopherol, or a γ-tocopherol-rich mixture reduced tumor volumes [123]. These results, considered together with the lack of protection in the HER2/neu-driven mammary tumor model, suggest that hormonal status and tumor subtype may influence the effectiveness of dietary bioactive compounds.

Potential chemopreventive effects should be distinguished from the treatment of established tumors. In male rats exposed to AOM, dietary squalene at 1% reduced the formation of colonic aberrant crypt foci by more than 46%. Because aberrant crypt foci are preneoplastic lesions [136], these findings support a potential effect on early colorectal carcinogenesis but do not establish a reduction in invasive cancer or therapeutic efficacy against established tumors. Further, β-sitosterol has demonstrated activity in both cellular and animal models. In human colon adenocarcinoma cells, it inhibited cell growth and reduced the expression of β-catenin and proliferating cell nuclear antigen. In colon carcinogenesis rats, β-sitosterol administration reduced aberrant crypt numbers and crypt multiplicity in a dose-dependent manner [136]. Further studies are required to determine the mechanisms underlying these effects and to establish whether comparable exposures can be achieved in humans.

Promising research directions include comparing the effectiveness and mechanisms of individual bioactive compounds, identifying the cancer subtypes most likely to respond, and determining whether their effects on apoptosis and cell-cycle progression selectively target malignant cells. Further studies should characterize concentration–response relationships, investigate the context-dependent effects of these compounds on ROS production, antioxidant defenses, and inflammatory signaling, and distinguish direct effects on cancer cells from indirect effects mediated by the tumor microenvironment. In addition, their potential to inhibit migration, invasion, metastasis, and angiogenesis should be evaluated in cancer-specific animal models, with separate assessment of primary tumor growth, metastatic dissemination, and established metastases. Identifying predictive biomarkers and clarifying the molecular determinants of treatment sensitivity could help elucidate which compounds are most promising for particular tumor types. An additional important priority is to investigate whether selected compounds can enhance responses to conventional anticancer treatments, including endocrine therapy, chemotherapy, and targeted agents. Finally, pharmacokinetic, bioavailability, and safety studies are essential to determine whether effective experimental concentrations can be achieved in vivo and whether these compounds have sufficient therapeutic selectivity. Collectively, these investigations should account for tumor heterogeneity and differences between experimental models before progression to clinical trials, as the current preclinical evidence does not yet establish their efficacy or safety as anticancer interventions in humans.

Future research should address existing gaps in clinical applications by prioritizing the use of well-characterized, standardized EVOO preparations and enhancing the precise quantification of individual phenolic compounds and their metabolites. Additional pharmacokinetic studies are also required to elucidate bioavailability, tissue distribution, metabolic pathways, and physiologically relevant concentrations. Transitioning from traditional 2D cell cultures to 3D spheroids, organoids, and appropriate animal models may yield evidence with greater clinical applicability. Another critical part involves investigating combinations of olive oil bioactive compounds with established cancer drugs to identify synergistic effects or chemosensitization. The potential of such combinations to mitigate treatment-related toxicity warrants further investigation. Finally, rigorously designed clinical studies are essential to determine whether the biological effects observed in experimental models translate into tangible benefits for cancer prevention or supportive care. These studies should employ standardized preparations, clearly defined dosing regimens, appropriate biomarkers, and robust assessments of safety and clinical outcomes.

## 8. Limitations and Priorities for Clinical Translation

The evidence remains predominantly preclinical and varies markedly in strength across the compounds, mechanisms, and cancer models examined. In vitro studies are useful for identifying molecular responses but cannot demonstrate the complexity of tumor properties, immune interactions, drug distribution, metabolism, and toxicity in humans. Moreover, concentrations effective in vitro may not be reachable in tissues following dietary exposure. High experimental concentrations, differences in treatment duration, and the use of purified compounds or enriched extracts further complicate associations across studies. Animal experiments also have important limitations, including small sample sizes, differences between transplanted tumors and naturally arising cancers, and variation in dose and route of administration. A reduction in cell viability, preneoplastic lesions, tumor volume, or angiogenic activity represents a distinct result; these endpoints should not be considered interchangeable evidence of cancer prevention, metastatic control, or prolonged survival. In addition, experiments involving olive-leaf extracts, phenolic mixtures, or whole olive oil cannot establish the independent contribution of an individual constituent unless the intervention has been adequately characterized. Human evidence remains insufficient to establish the anticancer efficacy of these compounds. Dietary studies involving EVOO cannot determine whether observed outcomes are attributable to a specific compound, the overall dietary pattern, other oil constituents, or confounding factors. Likewise, experimental doses administered to animals should not be translated directly into recommended human doses without pharmacokinetic, toxicological, and dose-finding studies. Future research should prioritize chemically characterized preparations, reproducible dose–response studies, pharmacokinetic and bioavailability assessment, cancer-specific animal models, and direct comparisons with standard treatments. Mechanistic studies should establish causal targets rather than relying solely on changes in protein expression or pathway-associated biomarkers. Carefully designed early-phase clinical trials will subsequently be required to assess safety, tolerability, achievable exposure, and biological activity, followed by controlled trials with sufficient statistical power to evaluate clinically significant outcomes.

## Figures and Tables

**Table 1:** Chemical composition of olive oil.

Fraction	Component Group	Principal Constituents	Typical Concentration
Saponifiable fraction (>98%)	Monounsaturated fatty acids	Oleic acid	55–83% of total fatty acids
	Saturated fatty acids	Palmitic acid	7.5–20%
	Polyunsaturated fatty acids	Linoleic acid	3.5–21%
Unsaponifiable fraction (<2%)	Phenolic compounds	Hydroxytyrosol, Tyrosol	mg/kg range
	Secoiridoids	Oleuropein, Oleacein, Oleocanthal	mg/kg range
	Hydrocarbons	Squalene	200–7500 mg/kg
	Tocopherols	α-Tocopherol (Vitamin E)	tens–hundreds mg/kg
	Sterols	β-sitosterol	hundreds–thousands

Note: This table was constructed based on data reported in the reference [47].

## Data Availability

The data presented in this study are available on request from the corresponding author.
